# Recent advances in *Bletilla striata* polysaccharide research: extraction methodologies, structural elucidation, pharmacological mechanisms, structure–activity relationships, and therapeutic delivery applications

**DOI:** 10.3389/fphar.2025.1688676

**Published:** 2025-11-05

**Authors:** Bo Wang, Chaomeng Wu, Yingjuan Ma, Xiaofen Liu, Lijun Tao, Xuebing Zhou, Limin Jia

**Affiliations:** ^1^ People’s Hospital of Ningxia Hui Autonomous Region, Ningxia Medical University, Yinchuan, China; ^2^ Department of Endocrinology, The Third Clinical Medical College of Ningxia Medical University, Yinchuan, China

**Keywords:** *Bletilla striata*, polysaccharide, bioactivity, structure–activity, relationships, drug carrier

## Abstract

*Bletilla striata* is a traditional Chinese medicine (TCM) used for hemostasis, detumescence, and tissue regeneration. Its major bioactive component, *Bletilla striata* polysaccharide (BSP), is a water-soluble heteropolysaccharide composed primarily of mannose and glucose. Various extraction techniques—including hot water, ultrasonic assisted, and microbial fermentation methods—have been developed to isolate BSPs, with extraction parameters significantly influencing its structural features. BSPs exhibit diverse pharmacological activities, such as wound healing, immunomodulation, anti-inflammatory, antioxidant, and gut microbiota regulatory effects. Despite extensive studies, the structure–activity relationships (SARs) and toxicological profile of BSPs remain incompletely understood. Owing to its favorable biodegradability and biocompatibility, BSPs show promise as a nanocarrier for drug delivery. This review summarizes advanced purification and structural characterization techniques, pharmacological mechanisms, potential toxicities, and drug delivery applications of BSPs, providing a translational framework for future research and development.

## 1 Introduction

**FIGURE 1:**
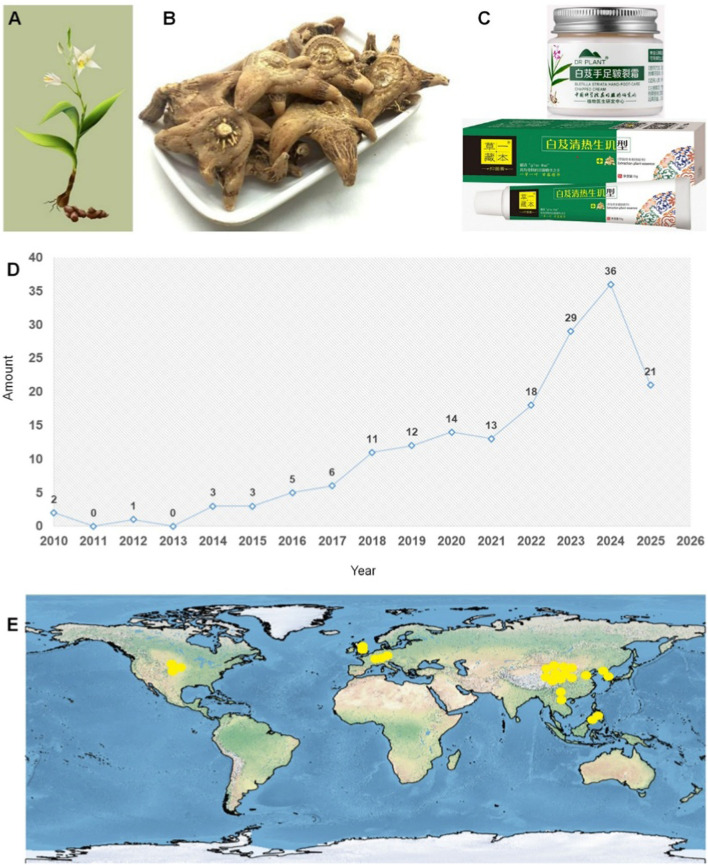
Morphological characteristics of *B. striata* **(A)**; the rhizome of *B. striata* **(B)**; commercially available *B. striata* products **(C)**; trends in publications related to BSPs (2010–2025), data from Web of Science (https://www.webofscience.com/), search term BSP **(D)**. Distribution of *B. striata* (SimpleMappr) **(E)**.

*Bletilla striata* (Thunb. ex A. Murray) Rchb. f. represents a perennial herbaceous species within the Orchidaceae family. Globally distributed, this species exhibits diverse vernacular nomenclature: in China, Baiji, Gangen, Zhulan, or Zilan; in Korea, Jaran; in Japan, Shiran; in Denmark, Mikodoblomst; in Sweden, Mikadoblomma; and in Germany, Japanorchidee (He et al., 2017). As illustrated in Figures 1A,B, the plant exhibits ornamental flowers and medicinal tubers containing bioactive compounds (Hu et al., 2024). It thrives in warm, humid, shaded environments but demonstrates low frost tolerance and photophobic characteristics (Luo et al., 2025). Its native range spans multiple Chinese regions (northwest, southeast, north, east, and central) (Xu et al., 2025), with a global distribution extending across East Asia (Japan, Korea) and Southeast Asia (Vietnam, Thailand, Myanmar) and introduced populations in Europe and North America (Bown, 2014; Wiart, 2012), as depicted in Figure 1E. Historically significant in traditional medicine, *B. striata* is classified among the “seven white” medicinal herbs in traditional Chinese medicine (TCM), first documented in *Shennong’s Classic of Materia Medica*. According to TCM theory, it possesses bitter, sweet, astringent, and slightly cold properties, offering astringent, hemostatic, swelling-reducing, and tissue-regenerative effects. It has been clinically applied to treat hemoptysis, hematemesis, traumatic hemorrhage, cutaneous ulcers, dermal toxicity, and xerosis. Modern pharmacological studies confirm that crude extracts and bioactive constituents of *B. striata* exhibit multifaceted bioactivities, including antioxidant (Song et al., 2017), anti-tumor (Sun et al., 2016), hemostatic (Venkatrajah et al., 2012), wound-healing (Song et al., 2017), antiviral (Shi et al., 2017), and antibacterial activities (Qian et al., 2015). Phytochemical research has identified over 158 distinct compounds in *B. striata*, including triterpenoids, saponins, steroidals, flavonoids, polysaccharides, polyphenols, and various other chemical constituents. Among these, *B. striata* polysaccharides (BSPs) demonstrate significant therapeutic potential, particularly in wound healing (Luo et al., 2010), hemostasis (Wang et al., 2006), antioxidation (Hu et al., 2024), anti-inflammatory (Diao et al., 2008), hepatoprotective (Jiang et al., 2023), anti-fibrotic (Wang et al., 2014), and immunomodulatory properties (Huang et al., 2024). These multifunctional attributes render BSPs highly valuable as biomaterials for advanced therapeutic platforms (Figure 1C), enabling applications in medicinal and skincare products.

BSPs are natural macromolecular polymers composed of >10 monosaccharide units connected via glycosidic bonds into linear or branched architectures, with molecular masses ranging from tens of thousands to millions of Daltons, predominantly consisting of mannose and glucose residues. Their favorable safety profile and minimal toxicity support broad biomedical applicability. Consequently, the significant therapeutic potential of BSPs has stimulated growing scientific interest, reflected in the increasing number of related publications (Figure 1D). However, increasing demand for *B. striata* in TCM has led to overharvesting and habitat loss, threatening its wild populations. Sustainable cultivation practices and conservation efforts are urgently needed to ensure the long-term availability of resources.

Natural polysaccharides such as BSPs exhibit advantageous physicochemical properties, such as water solubility, low toxicity, minimal immunogenicity, and ease of chemical modification, making them promising nanocarrier platforms for biomedical applications. Substantial evidence indicates that the bioactivity of BSPs is closely linked to structural features, including glycosidic linkage patterns, monosaccharide composition, and molecular conformation. Beyond their intrinsic pharmacological properties, BSPs serve as effective vehicles for targeted drug delivery. This comprehensive review examines the extraction methodologies, purification techniques, structural characteristics, pharmacological activities, and drug delivery applications of BSPs, laying a foundation for clinical translation and therapeutic development.

## 2 Botanical characterization

*B. striata* exhibits a mature height of 18–60 cm. Its rhizomes are characterized by a compressed, subglobose to irregular morphology, with diameters of 1–3 cm. The stems are robust, measuring 3–25 cm in length, and typically bear 4–6 leaves arranged in a widely spaced phyllotaxy. The inflorescence features a slender, gracefully arching peduncle measuring 14–34 cm in length, generally subtended by a single sheathing bract. The rachis is flexuous or pendulous, 2–7 cm long, and supports 3–10 flowers. The flowers are erect and conspicuous and exhibit a vivid purplish-red coloration. The sepals are purplish-red or pink, narrowly oblong in shape, with the lateral sepals displaying a sharply pointed apex and oblique orientation. The labellum is white, prominently veined with purplish-red, obovate-elliptical in outline, and medially trifid. The mid-lobe is distinctly quadrangular with crispate (finely wavy) margins and a truncate apex. The column is subterete (nearly cylindrical), slender, winged, and gradually dilated apically. Notably, a large rostellum serves as a key diagnostic feature. Plant characteristics of *B. striata* are shown in Table 1.

**TABLE 1:** Key identification characteristics of *B. striata*.

Plant part	Characteristic	Description
General habit	Mature height	18–60 cm
Rhizome	Morphology	Compressed, subglobose (almost spherical) to irregular
	Diameter	1–3 cm
Stem	Character	Robust
	Length	3–25 cm
Leaf	Number and arrangement	4–6 leaves; arranged in a widely spaced pattern (phyllotaxy)
Inflorescence	Peduncle (stalk)	Slender, gracefully arching
	Peduncle length	14–34 cm
	Rachis length	2–7 cm
	Flower number	3–10
Flower color	General appearance	Vivid purplish-red
Labellum (Lip)	Coloration	White with prominent purplish-red veins
Rostellum	Size and diagnostic value	A large, beak-like structure

## 3 Extraction and purification of *B. striata* polysaccharides

### 3.1 Extraction

**FIGURE 2:**
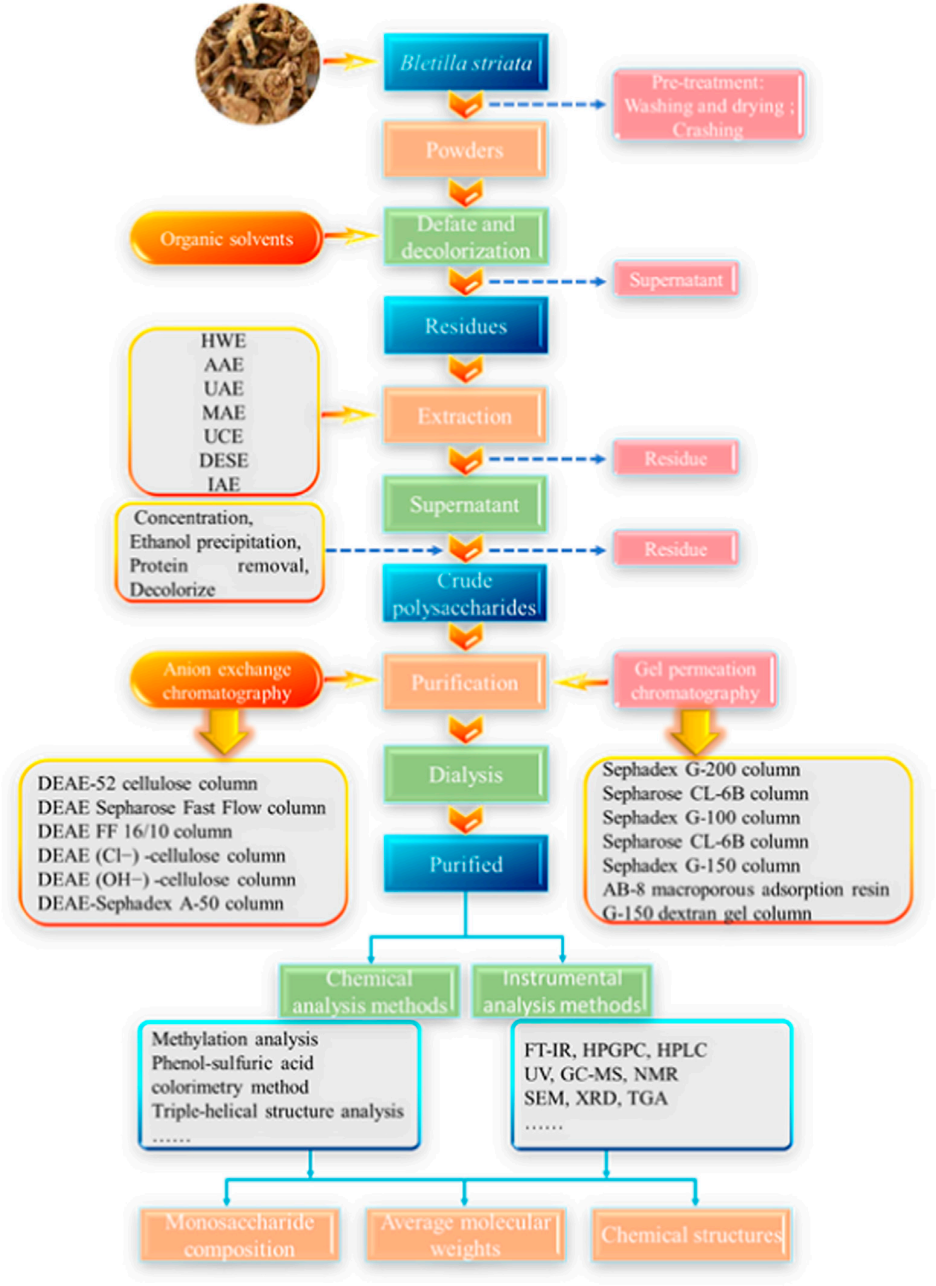
Schematic diagram of the extraction and purification procedures of polysaccharides isolated and purified from *B. striata*.

Currently, the most commonly reported methods for polysaccharide extraction from *B. striata* include hot water extraction (HWE), ultrasonic assisted extraction (UAE), and composite enzymatic hydrolysis. However, due to their distinct underlying mechanisms, each extraction method presents unique advantages and limitations (Table 2). The overall workflow for BSP extraction and purification is schematically illustrated in Figure 2. Key operational parameters, including extraction method, duration, temperature, solid-liquid ratio, final polysaccharide yield, and purification techniques, are systematically summarized in Supplementary Table 1.

**TABLE 2:** Comparison of extraction methods for *B. striata* polysaccharides.

Extraction method	Advantages	Disadvantages
Hot water extraction (HWE)	• Simple operation and low equipment cost • Environmentally friendly (water as solvent) • High safety and suitable for large-scale production • High extraction yields for hydrophilic polysaccharides	• Time-consuming and energy-intensive • High temperature may degrade heat-sensitive components • Low selectivity for specific polysaccharide types • Potential for polysaccharide degradation under prolonged heating
Acid-Base Extraction Method	• Effective for breaking down cell walls and extracting bound polysaccharides • Can enhance yield by hydrolyzing non-target components • Relatively simple process	• Harsh conditions may cause degradation of acid-/alkali-labile glycosidic bonds • Requires neutralization, generating salt waste • Corrosive to equipment and poses safety risks • May alternative polysaccharide structure and bioactivity
Microwave-assisted extraction (MAE)	• Rapid heating and significantly reduced extraction time • Higher yield and efficiency than HWE • Lower solvent consumption and energy usage • Improved selectivity and better preservation of bioactivity	• Requires specialized microwave-transparent equipment • Inhomogeneous heating may lead to hotspots and uneven extraction • Scaling up to industrial level can be challenging • Optimization of parameters (power, time, and temperature) is crucial
Ultrasonic assisted extraction (UAE)	• Enhanced mass transfer and cell wall disruption via cavitation • Lower operating temperatures, preserving thermolabile compounds • Reduced extraction time and solvent consumption • Simple setup and easy integration with other methods	• High energy consumption for large-scale applications • Potential degradation of polymers by prolonged ultrasonic energy • Process parameters (amplitude, frequency, and time) need optimization • Limited penetration depth in large volumes
Ultrasound-compound enzyme (UCE)	• Synergistic effect: enzyme specificity and ultrasonic physical disruption • High selectivity and yield under mild conditions (low temperature, neutral pH) • Minimal structural degradation and high bioactivity preservation • Environmentally friendly with reduced chemical usage	• High cost of enzymes • Enzymes require precise control of pH and temperature • Complex process optimization (enzyme selection and ultrasonic parameters) • Potential enzyme deactivation by ultrasound if not carefully controlled
Microbial fermentation extraction (MFE)	• Extremely mild conditions (ambient temperature/pH), ideal for labile compounds • High selectivity via enzymatic hydrolysis (proteases and glycosidases) • Potential for simultaneous extraction and modification, enhancing bioactivity • Eco-friendly and low energy consumption	• Time-consuming (requires days of fermentation) • High risk of microbial contamination requires strict sterility • Complex downstream purification from fermentation broth • Reproducibility challenges due to sensitivity to fermentation parameters
Deep eutectic solvent extraction (DESE)	• Tunable solubility by designing DES composition • High extraction efficiency and selectivity for polar compounds • Low volatility, non-flammability, and high biodegradability • Potential for designer green solvents with high sustainability	• High viscosity may limit mass transfer and require heating/stirring • Challenges in complete removal and recycling of DES from extract • Limited comprehensive data on toxicity of all DES components • Relatively high cost of some DES precursors compared to water
Infrared-assisted extraction (IAE)	• Rapid and uniform heating through radiation • Energy-efficient with deep penetration into biomass • Shorter extraction time than that of HWE • Can be combined with other methods for synergy	• Less developed and studied compared to MAE/UAE • Requires specialized infrared heating equipment • Risk of overheating and degrading surface compounds • Optimization parameters are not yet well-established

As the most widely utilized extraction technique, HWE isolates water-soluble polysaccharides from *B. striata* by heating distilled water to 50 °C–100 °C for 1.5–5 h. The initial pre-processing of *B. striata* tubers consists of washing, drying, and pulverizing them into small particles. This is commonly achieved via Soxhlet extraction using apolar solvents such as 95% ethanol or petroleum ether over several hours to degrease and decolorize the material (Chen et al., 2019). The residue is subsequently recovered and undergoes aqueous extraction at 90 °C with constant agitation for multiple hours. The differential solubility of polysaccharides in water and ethanol facilitates their recovery through stepwise precipitation using water and ethanol at different concentration ratios. In standard HWE protocols, Wang Q. et al. (2023) reported a 20% (w/w) BSP yield using HWE with ethanol precipitation. Subsequent optimization through orthogonal experimental design identified extraction time and water-to-solid ratio as dominant yield factors, achieving a 40.99% (w/w) BSP yield under optimal conditions (67.79 °C, 102 min, solid–liquid ratio 1:59.77 (w/v)) with three extraction cycles (Zifan Wang and Zhang, 2022). Despite these benefits, HWE exhibits significant limitations: (1) co-extraction of organic acids and anthraquinones compromises BSP purity and bio-functional properties; (2) limited polysaccharide solubility in hot water necessitates supplementary solubilization strategies; (3) thermal degradation at high temperatures reduces bioactivity and functional stability; (4) time-intensive processing impedes efficiency, particularly for structurally complex BSP fractions; and (5) inherent inefficiencies (low yield, high energy consumption) limit industrial scalability.

The recovery yield and purity of polysaccharides can be significantly improved by modifying conventional hot water extraction with the addition of optimized concentrations of alkaline (NaOH or KOH) or acidic (TFA or HCl) reagents. Acidic conditions enhance the dissolution of polysaccharides from the plant cell wall matrix, while alkaline media facilitate solubilization through hydrogen bond cleavage and disruption of protein–polysaccharide complexes. A recent systematic optimization study assessed four critical alkaline extraction parameters: NaOH concentration, temperature, NaOH-to-solid ratio, and duration. Chen H. et al. (2023) employed response surface methodology (RSM) integrated with genetic algorithm–artificial neural network (GA–ANN) modeling, identifying optimal conditions as follows: 52 °C, 1:30 solid–liquid ratio (w/v), 167 min duration, and 0.01 mol/L NaOH, yielding 29.53% BSP recovery. Conversely, some studies report reduced polysaccharide yields with dilute alkali compared to pure HWE (Chen H. et al., 2021), likely attributable to glycosidic bond scission and partial hydrolysis in acidic/alkaline media, which compromise structural integrity. For acidic extraction, Wang et al. (2023a) established parameters for BSP: 1:9 material-to-liquid ratio, 90 °C, and 90 min duration. Compared to thermal hydrolysis alone, acid/alkaline-assisted extraction offers enhanced efficiency, lower energy input, and improved reproducibility. However, stringent parameter control increases process complexity and substantially elevates downstream processing costs. Additionally, alkaline extraction imparts darkened coloration and persistent alkaline residues, degrading product quality. Despite limitations in yield, prolonged processing times, and purification challenges, aqueous extraction remains prevalent (Supplementary Table 1) due to straightforward implementation and economic viability.

As an advanced extraction technique, microwave-assisted extraction (MAE) employs electromagnetic radiation to trigger instantaneous internal heating through molecular dipole rotation. This energy absorption causes molecular friction and ionic migration (Li J. F. et al., 2025), effectively rupturing plant cellular matrices and enhancing the diffusion of target compounds, such as BSPs, into the extraction solvent. For BSP extraction specifically, Han et al. (2025) established the MAE extraction method to extract BSPs. The optimal conditions were as follows: solid–liquid ratio, 1:30; extraction time, 9 min; microwave power, 600 W; and yield, 42.82% ± 1.87%. However, MAE implementation faces practical constraints: high capital investment for industrial-scale systems; limited applicability for thermolabile compounds due to localized superheating; and technical complexity in scaling batch processes.

Ultrasonic assisted extraction (UAE) leverages the mechanical effects of ultrasound-induced cavitation to enhance interfacial mass transfer, enabling uniform disruption of *B. striata* cell walls and thereby accelerating BSP release, diffusion, and dissolution for improved yield and efficiency. As a non-thermal process, UAE’s efficiency is governed by five key parameters: solvent selection, solid-to-liquid ratio, ultrasonic power, temperature, and duration. Since distinct permutations of these factors alter constituent yields and material properties, identifying optimal conditions is essential to maximize productivity while minimizing energy consumption. Empirically, Qiu et al. (2024a) utilized UAE for BSP isolation, optimizing parameters via orthogonal design, Box–Behnken Design (BBD), and a genetic algorithm-backpropagation (GA-BP) neural network. The optimal conditions comprised a 15 mL/g liquid-to-solid ratio, 450 W ultrasonic power, and a 34-min duration, achieving an 8.29% (w/w) crude BSP yield. In another study, Han et al. (2025) demonstrated UAE’s superiority over HWE under identical conditions, reporting enhanced extraction efficiency accompanied by reduced BSP molecular weights. These studies confirm UAE as a scalable technique readily optimized for industrial-scale applications. However, UAE exhibits significant limitations, including substantial equipment costs and energy demands; thermal degradation risks during prolonged operation; cavitation-induced structural alterations (Han et al., 2025), reducing molecular weight (Chen H. et al., 2021) and altering spatial conformation (Wang et al., 2023c); and consequent bioactivity impairment through diminished solution viscosity, purity, and functional integrity (Afshari et al., 2015; Li S. et al., 2022); and scalability constraints in continuous processing systems.

Ultrasonic irradiation enhances enzyme-substrate affinity and accelerates hydrolysis kinetics by reducing mass transfer barriers and increasing molecular collision frequency. Building upon established ultrasonic-assisted and enzymatic protocols, we developed a synergistic ultrasound compound enzyme (UCE) method for BSP isolation. Optimized parameters included high-temperature amylase and 0.1 g acid protease enzyme dosage, 70 °C extraction temperature, a 1:30 solid–liquid ratio, and 30 min ultrasonication, achieving a 66.37% polysaccharide yield (Huang et al., 2024). Structural analysis revealed UCE-extracted BSP as a novel neutral glucomannan featuring mannose and glucose subunits, low molecular weight, and aqueous solubility. Consequently, UCE demonstrates significant potential for pharmaceutical applications, particularly for thermolabile bioactive polymers requiring structural preservation.

The primary objective of microbial fermentation is to harness the organic acids or proteolytic enzymes generated by microbial activity to facilitate polysaccharide extraction. The literature indicates that polysaccharide extraction through microbial fermentation utilizes various microorganisms, which can be classified into acid-producing and protease-producing groups. Microbial fermentation extraction (MFE) represents an advanced green technology that leverages controlled microbial metabolism to enhance polysaccharide bioactivity while reducing environmental impact, positioning it as a promising industrial-scale approach. However, this method faces several challenges: microbial strain specificity, enzyme-substrate compatibility, and fermentation condition dependency. For instance, Shu et al. (2017) demonstrated that *B. striata* polysaccharides selectively promoted the proliferation of specific probiotic strains (e.g., L. acidophilus LA-5, B. bifidum BB01, and L. bulgaricus LB6), indicating strain-specific utilization efficiency of polysaccharides. Additionally, Yang et al. (2021) highlighted that enzymes secreted by different strains (e.g., *Aspergillus niger* and *Thermoascus aurantiacus*), such as endo-xylanases and β-glucanases, exhibited significantly varied hydrolysis efficiencies toward non-starch polysaccharides (e.g., arabinoxylan and β-glucan), directly impacting polysaccharide extraction yields and product composition. Furthermore, Shu et al. (2017) optimized fermentation conditions via response surface methodology and found that critical parameters (e.g., pH 7.79 and enzyme concentration 2.73%) must be precisely matched to strain characteristics to avoid significant reductions in extraction efficiency. Moreover, Dora Elisa Cruz-Casas emphasized that batch-to-batch reproducibility in microbial fermentation is often poor due to sensitivity to environmental fluctuations (e.g., pH, temperature, and pressure), leading to inconsistent product quality during scale-up. For example, lactic acid bacteria fermentation may introduce metabolic byproducts (e.g., antimicrobial agents and exogenous polysaccharides) that compromise target polysaccharide purity. Finally, although microbial fermentation offers cost advantages (e.g., no need for commercial enzymes), Dora Elisa Cruz-Casas noted that the difficulty of separating and purifying byproducts increases with scale, potentially offsetting these economic benefits. Through optimized cultivation parameters, MFE enables targeted BSP extraction via enzymatic hydrolysis and bioconversion.

Empirical validation comes from Wang Q. et al. (2024), who employed *Bacillus* licheniformis for one-step fermentation extraction of FBP, establishing optimal parameters: 12 h fermentation time, pH 6.25, 5% inoculum size, and 37 °C temperatures. These optimized conditions achieved a 33.77% (w/w) FBP extraction yield, and the purity of FBP was 90.82%. Studies revealed reduced molecular weight and viscosity of FBP during microbial fermentation, concurrent with substantial solubility improvement. Comprehensive evidence confirms that microbial fermentation alters polysaccharide characteristics while augmenting biological activity. Despite these advantages, MFE implementation faces challenges, including technical complexity in strain selection and process control, high capital costs, specialized knowledge gaps in microbial physiology, and limited scalability data for industrial translation. Addressing these challenges requires cross-disciplinary research integrating microbiology and process engineering; bioreactor optimization for energy efficiency; industrial–academic partnerships to bridge laboratory-to-pilot gaps; and techno-economic analysis to validate commercial viability. Strategic advancement of MFE could revolutionize plant polysaccharide production, establishing sustainable extraction paradigms for the biopharma sector.

As a novel class of green solvents, deep eutectic solvents (DESs) have attracted considerable research interest owing to their fundamentally distinct physicochemical properties, establishing them as viable and sustainable replacements for traditional organic solvents. The environmental benefits of DESs are exemplified by their utilization of naturally derived, low-toxicity components, low-energy manufacturing protocols, negligible volatility, and complete avoidance of volatile organic compound (VOC) releases. Moreover, specific DES systems (such as choline chloride/glycerol formulations) exhibit superior biodegradability profiles, achieving degradation efficiencies surpassing 95%, which markedly reduces potential environmental accumulation hazards (Schuh et al., 2023). DESs are regarded as a new generation of green solvents due to their unique physicochemical properties and environmental friendliness, demonstrating significant advantages in the field of plant polysaccharide extraction (Feng et al., 2023). A growing body of research indicates that DESs have gained widespread applications in bioactive compound extraction, consistently demonstrating superior extraction efficiency over conventional solvents. This conclusion directly corroborates the findings of Shafie et al. (2019), who utilized a choline chloride–citric acid monohydrate-based DES for polysaccharide compound extraction. The results demonstrated a significantly enhanced polysaccharide yield with DESs (14.44%), substantially outperforming the conventional solvent, citric acid monohydrate, which yielded only 9.34%. In a pioneering study, Luo et al. (2023) extracted BSPs from *B. striata* using the DESE method. They optimized the extraction process using single-factor and Box–Behnken response surface tests to determine the parameters presented below: extraction time of 47 min, extraction temperature of 78 °C, liquid-to-solid ratio of 25 mL/g, and water content of 35%. Under these conditions, BSP-2 yield reached 558.90 ± 8.83 mg/g, representing a 2.7-fold increase over conventional hot water extraction. This performance enhancement stems from DESs’ ability to disrupt hydrogen-bonding networks within plant cell walls while preserving polysaccharide structural integrity.

Recent advances in polysaccharide extraction have introduced auxiliary techniques such as infrared-assisted extraction (IAE). This method leverages preferential energy absorption within the infrared spectrum (2.5–100 µm) to generate molecular vibrations that enhance heat transfer efficiency while minimizing energy dissipation through radiative heating (Chen et al., 2010). The core mechanism involves infrared radiation inducing rapid thermal excitation, which accelerates solvent diffusion into plant matrices and promotes target compound dissolution, thereby improving extraction efficiency and reducing processing duration (Xiang B. et al., 2022). Optimization studies employing single-factor design, BBD, and response surface methodology achieved a BSP yield of 43.95% ± 0.26% under IAE (Qu et al., 2016), representing a 19% increase over conventional hot water extraction.

In summary, diverse extraction protocols have been established for BSPs, with methodological selection critically determining crude polysaccharide recovery rates. In contrast, alkaline-mediated extraction represents a highly effective method for dramatically increasing polysaccharide yields. Furthermore, advanced methodologies (UAE, EAE, MAE, MFE, DESE, and IAE) collectively optimize BSP isolation through yield amplification, process intensification, and substantial temperature reduction.

### 3.2 Separation and purification of *B. striata* polysaccharide

Ethanol precipitation exploits the differential solubility of polysaccharides, which are highly soluble in water yet insoluble in polar organic solvents, by reducing polysaccharide solubility through ethanol addition, thereby inducing selective precipitation. Gradient ethanol precipitation further exploits the inverse correlation between polysaccharide molecular weight and ethanol precipitation concentration: higher molecular weight fractions precipitate at lower ethanol concentrations, enabling molecular weight-based fractionation. Lai et al. (2018) purified crude BSP extract through ethanol precipitation using three volumes of 95% (v/v) ethanol, followed by overnight incubation at 4 °C, yielding polysaccharides with a molecular weight of 1.98 × 10^5^ Da. In addition, Liu et al. (2022a) isolated antitumor-active low molecular weight polysaccharide (LMW-BSP, 23 kDa) through sequential ethanol fractionation: freeze–thaw-concentrated aqueous extract was first precipitated with 50% (v/v) anhydrous ethanol (12 h, 4 °C), followed by centrifugation and ethanol supplementation to 70% (v/v) final concentration.

Post-extraction polysaccharides typically contain proteinaceous and chromophoric contaminants that compromise structural characterization and bioactivity evaluation. Since proteins and polysaccharides share polar macromolecular properties, targeted protein removal is essential. Current deproteinization techniques include Sevag treatment, freeze-thaw cycling, TCA precipitation, enzymatic-Sevag hybrids, and enzyme-assisted freeze–thaw cycling (Table 1). Among these, the Sevag method using a 4:1 chloroform: n-butanol mixture remains widely applied for protein elimination from BSPs through selective partitioning of uncomplexed proteins (Li et al., 2021). Nevertheless, Sevag demonstrates operational inefficiency, leaves persistent toxic contaminants, and often results in considerable macromolecule depletion. Subsequent research must establish innovative approaches with improved efficacy and reduced ecological footprint. Consequently, advanced methods such as combined enzymatic-freeze–thaw treatment demonstrate superior performance. Hou et al. (2023) achieved 99.3% protein removal by incubating crude polysaccharide solution with thermostable α-amylase (3000 U/mL) at 70 °C, followed by three freeze–thaw cycles.

Simultaneously, pigment-laden polysaccharides must undergo decolorization to prevent chromatographic media fouling. Activated carbon adsorption serves as the standard method, leveraging its high surface area (500–1,500 m^2^/g) to adsorb pigments via hydrophobic interactions (Cui et al., 2017). Following deproteinization and decolorization, residual low-molecular-weight contaminants (e.g., inorganic salts) require removal through extended dialysis or high-speed centrifugation. These purification steps collectively enhance BSP purity for downstream applications.

Crude BSP preparations obtained through prior extraction and purification steps require further refinement via chromatographic techniques to achieve pharmaceutical-grade purity. Standard protocols employ a sequential approach, including ion-exchange and gel-permeation chromatography. In anion-exchange chromatography, polysaccharides are separated based on charge-density differences through adsorption-partition mechanisms. Common stationary phases include the DEAE-52 cellulose column, DEAE Sepharose Fast Flow Gel column, DEAE FF 16/10 column, DEAE (Cl^−^)-cellulose column, DEAE (OH^−^)-cellulose column, and DEAE-Sephadex A-50 column (Zhang R. et al., 2022). Gel column chromatography functions as a molecular sieve, separating polysaccharides based on hydrodynamic volume through size-exclusion mechanisms. Effective media comprise the following: Sephadex G-200 column, Sepharose CL-6B column, Sephadex G-100 column, Sephadex G-150 column, AB-8 microporous adsorption resin, and G-150 dextran gel column (Jia et al., 2025).

This orthogonal purification strategy typically achieves over 90% polysaccharide purity. However, inherent limitations persist, including irreversible adsorption losses (5%–15% polysaccharide retention on stationary phases), suboptimal elution efficiency during packing/loading cycles, and mobile-phase mismatch leading to incomplete analyte recovery. Additionally, the surface chemistry of stationary phases (including ligand density), mobile phase ionic strength/pH, and elution gradient design constitute key factors affecting yield.

## 4 Structural characteristics

Polysaccharides are macromolecular polymers composed of over 10 monosaccharide units linked via glycosidic bonds. Their structural features, including monosaccharide composition, molecular weight distribution, glycosidic linkage patterns, stereochemical configurations, and anomeric conformations, critically determine biofunctional properties. Currently, over 60 distinct BSP fractions have been isolated from *B. striata* tissues (Table 2), with structural heterogeneity primarily attributable to methodological variations in extraction and purification protocols. Minor structural alterations can profoundly impact bioactivity, necessitating systematic characterization to establish robust structure–activity relationships (SARs). To comprehensively elucidate BSP structures, we employed an integrated analytical platform: UV–visible spectroscopy, gas chromatography (GC), high-performance liquid chromatography (HPLC), size-exclusion chromatography (SEC, including high-performance gel permeation chromatography (HPGPC) variants), GC–mass spectrometry (GC-MS), FT-IR spectroscopy, multinuclear NMR (^1^H, 13C, and 2D), atomic force microscopy (AFM), scanning electron microscopy (SEM), and X-ray diffraction (XRD). Table 3 comprehensively catalogs each polysaccharide’s nomenclature, structural characterization methods, monosaccharide composition (molar ratios), molecular weight distribution, key glycosidic linkages, and primary literature references.

**TABLE 3:** Structural characteristics of the purified polysaccharide from *B. striata*.

Compound name	MW (kDa)	Monosaccharide composition (mole ratio)	Analysis method	Chemical structure	Types (*in* *vitro* or *in* *vivo*)	Biological activities	Reference
BSP-H	282.91	Glu: Man: Gal = 37.89:60.78:1.32	HPGPC, FT-IR, nanoparticle analyzer SZ-100Z, Congo red, GC–MS, NMR, X-ray	1,4-β-mannopyranose and 1,4-β-glucopyranose	*In* *vitro*	Antioxidant activity	Chen et al. (2021a)
BSP-B	230.63	Glu: Man: Gal = 47.55:51.30:1.16	HPGPC, FT-IR, nanoparticle analyzer SZ-100Z, Congo red, GC–MS, NMR, X-ray	1,4-β-mannopyranose and 1,4-β-glucopyranose	*In* *vitro*	Antioxidant activity	Chen et al. (2021a)
BSP-A	402.17	Glu: Man: Gal = 43.07:55.91:1.02	HPGPC, FT-IR, nanoparticle analyzer SZ-100Z, Congo red, GC–MS, NMR, X-ray	1,4-α-glucopyranose, 1,4-β-mannopyranose and 1,4-β-glucopyranose	*In* *vitro*	Antioxidant activity	Chen et al. (2021a)
BSP-U	195.83	Glu: Man: Gal = 38.05:61.04:0.92	HPGPC, FT-IR, nanoparticle analyzer SZ-100Z, Congo red, GC–MS, NMR, X-ray	1,4-β-mannopyranose and 1,4-β-glucopyranose	*In* *vitro*	Antioxidant activity	Chen et al. (2021a)
BSP	298.82	Glu: Man: Gal = 27.26: 71.81: 0.3	HPLC, XRD, NMR	α-D-Glcp-(1→, →4)-2-O-acetyl-β-D-Manp-(1→, →4)-β-D-Manp-(1→, →4)-β-D-Glcp-(1→)	*In vitro*	Antioxidant activities	Chen et al. (2024a)
DBSP-1	153.94	Glu: Man = 18.65: 81.35	HPLC, XRD, NMR	α-D-Glcp-(1→, →4)-2-O-acetyl-β-D-Manp-(1→, →4)-β-D-Manp-(1→	*In vitro*	Antioxidant activities	Chen et al. (2024a)
DBSP-5	66.96	Glu: Man = 21.16: 78.84	HPLC, XRD, NMR	α-D-Glcp-(1→, →4)-2-O-acetyl-β-D-Manp-(1→, →4)-β-D-Manp-(1→	*In vitro*	Antioxidant activities	Chen et al. (2024a)
DBSP-10	15.54	Glu: Man = 19.74: 80.26	HPLC, XRD, NMR	α-D-Glcp-(1→, →4)-2-O-acetyl-β-D-Manp-(1→, →4)-β-D-Manp-(1→	*In vitro*	Antioxidant activities	Chen et al. (2024a)
pFSP	91.00	Gal: Glu: Man = 2.03:1.00:3.45	HPGPC, HPLC, FT-IR, NMR, SEM	(1→4)-linked-α-D-Glcp, (1→4)-linked-β-D-Manp and (1→3,6)-linked-β-D-Manp units, together with the branches of (1→6)-linked-β-D-Galp and terminated with (1→)-linked-β-D-Manp residue	*In vitro*	Antioxidant activity	Chen et al. (2020a)
BSP-A	305.95	Man: Glu: Ara: Gal: GalA = 69.15: 27.89: 0.46: 0.69: 1.81	HPGPC, HPLC, Dionex ICS-3000, FT-IR, SEM	Triple helix conformation	*In vitro*	Antioxidant activity	Chen et al. (2023a)
pBSP	327.60	Glu: Man = 1: 1.34	HPGPC, HPLC, FT-IR, SEM, NMR	(1→4)-linked-β-D-Manp, (1→4)-linked-α-D-Glcp and (1→3)-linked-β-D-Manp	*In vitro*	Antioxidant capacity	Xu et al. (2021)
BSP-1	269.12	Glu: Man: Gal = 24.91: 74.56: 0.53	HPGPC, FT-IR, SEM, GC–MS, NMR	α-D-Glcp, β-D-Glcp, β-D-Manp, and 2-O-acetyl-β-D-Manp, with the branched-chain accompanied by β-D-Galp and α-D-Glcp	*In vivo*	Antioxidant capacity and anti-melanogenesis effects	Chen et al. (2024b)
BSP-2	57.39	Glu: Man: Gal: Ara = 26.06: 71.83: 1.81: 0.31	HPGPC, FT-IR, SEM, GC–MS, NMR	N/A	*In vivo*	Antioxidant capacity and anti-melanogenesis effects	Chen et al. (2024b)
BSP-3	28.15	Glu: Man: Gal: Ara: Rha = 24.03: 58.84: 9.84: 2.40: 4.89	HPGPC, FT-IR, SEM, GC–MS, NMR	N/A	*In vivo*	Antioxidant capacity and anti-melanogenesis effects	Chen et al. (2024b)
BSP-1	761.12	Man: Rib: Rha: GluA: Glu: Gal: Xyl: Fuc = 22.34:0.24:0.23:0.89:27.84:1:00:1.28:0.56	UV−VIS, FT-IR, X-ray, HPLC	N/A	*In* *vitro*	Immunomodulatory	Zhai et al. (2021)
BSP-2	843.94	Man: GalA: Glu: Gal = 52.32:2.44:31.26:1:00	UV−VIS, FT-IR, X-ray, HPLC	N/A	*In* *vitro*	Immunomodulatory	Zhai et al. (2021)
BSP-3	950.30	Man: Rha: GalA: Glu: Gal = 3.20:1.20:0.87:8.77:1.00	UV−VIS, FT-IR, X-ray, HPLC	N/A	*In* *vitro*	Immunomodulatory	Zhai et al. (2021)
BSP-4	715.46	Man: Rib: Rha: Glu: Gal: Xyl: Ara = 10.35:1.00:1.18:1.21:6.12:1.00:1.06:0.62	UV−VIS, FT-IR, X-ray, HPLC	N/A	*In* *vitro*	Immunomodulatory	Zhai et al. (2021)
BSP-1	83.54	Man: Glu: 4.0:1.0	UV, LC-6AD HPLC, HPGPC, GC, FT-IR, GC–MS, NMR	Repeating β-1,4-linked D-mannosyl residues and β-1,4-linked D-glucosyl residues	*In vivo*	Immunomodulatory activity	Wang et al. (2019b)
BSP-2	12.60	Man: Glu = 3.0:1.0	UV, LC-6AD HPLC, HPGPC, GC, FT-IR, GC–MS, NMR	Repeating β-1,4-linked D-mannosyl residues and β-1,4-linked D-glucosyl residues	*In vivo*	Immunomodulatory activity	Wang et al. (2019b)
BSP	28.37	Glu: Man = 1: 2.9	GPC, HPLC, GC-MS, NMR	→ 6)-β-Manp-(1→, →4)-β-Glcp-(1→, →4)-β-Manp-(1 → and →3)-α-Manp-(1 → linear main chain, containing β-Glcp-(1 → and β-Manp-(1 → two branched chain fragments were connected to the Man residue at position 4	*In vitro* and *in vivo*	Immunomodulatory effect	Huang et al. (2024)
BSP	48.30	Glu: Man = 2:1	HPGPC, SEM, FT-IR, GC–MS, NMR	Man: 1, 3, 6-linked, 1, 3-linked, and Glu was terminal-linked	*In vivo*	Trained immunity	Zhang et al. (2025)
pBSP1	255.17	Glu: Man = 1:2.95	SEM, UV, FT-IR, HPLC, XRD, NMR	1→ 6, 1→ 2, 1→ 4 glycosidic bonds and possibly 1 → 3 glycosidic bonds	*In vitro*	Intestinal protection activity	Qiu et al. (2024a)
BSP	170.00	Glu: Man = 2.55: 7.45	HPLC, FT-IR	N/A	*In vitro* and *in vivo*	Gastroprotective activity	Wang et al. (2020)
BP	221.17	Glu: Man = 23.81: 76.19	HPGPC, GC–MS, FT-IR, NMR	1,4-β-Mannopyranose, 1,4-β-glucopyranose, and a small amount of 1,4-α-glucopyranose	*In vitro*	Regulating gut microbiota	Wang et al. (2023a)
BO	0.94	Glu: Man = 22.22: 77.78	N/A	N/A	*In vitro*	Regulating gut microbiota	Wang et al. (2023a)
FBP	6.79	Glu: Man = 1:2.7	HPGPC, HPLC, FT-IR, GC–MS, NMR, SEM	β-1,4-linked mannose and β-1,4-linked glucose	*In vitro*	Prebiotic activity	Wang et al. (2024b)
BSP	58.50	Glu: Man = 2.6:7.4	HPGPC, FT-IR, GC–MS, NMR	→4)-β-D-Glcp-(1 → 4)-β-D-Manp-(1 → 4)-β-D-2aceManp-(1 → 4)-β-D-Manp-(1 → 4)-β-D-Glcp-(1 → 4)-β-D-Manp-(1 → 4)-β-D-Manp-(1 → 4)-β-D-3ace-Manp-(1→	*In vivo*	Anti-liver fibrosis	Jiang et al. (2023)
BSP	50.06	Glu: Man = 3.1: 6.9	HPGPC, UV, FT-IR, HPLC, NMR, SEM	Pyranose linked by α- and β-type glycosidic bonds	*In vitro* and *in vivo*	Pulmonary fibrosis	Wang et al. (2024c)
BPSb	260.00	Glu: Man = 1: 3	HPGPC, HPLC, FT-IR, NMR	(1 → 2)-linked α-D-mannopyranose and (1 → 4)-linked β-D-glucopyranose residues	*In vitro*	Anti-fibrosis effects	Wang et al. (2014)
BSP	290.75	Man (45.73%), Glu (23.75%), and Fru (15.31%), with minor amounts of Ara (12.56%), Gal (1.57%), and Rha (1.08%)	HPGPC, IC, GC-MS, FT-IR	Pyranose-type glucan, likely a glucomannan, with α-glycosidic linkages	*In* *vitro* and *in vivo*	Alleviate ARDS	Wu et al. (2025)
BSP	536.79	Glu: Man = 1.00: 1.84	HPGPC, UV, FT-IR, HPLC	N/A	*In vivo*	Promotes diabetic wound healing	Zhao et al. (2021)
BSPS	722.90	Glu: Man = 1: 2.5	HPGPC, GC-MS, FT-IR, NMR, SEM	→4)-β-D-Glcp-(1→ and →4)-β-D-Manp-(1→ residues. O-acetyl group linked to C2 of →4)-β-D-Manp-(1→ residue	*In vitro* and *in vivo*	Wound-healing	He et al. (2024b)
BSP-1	22.65	Glu: Man = 1: 3.68	HPGPC-RID-MALLS, HPLC, FT-IR, GC–MS, NMR	→4)-β-D-Man-(1→, →4)-α-DMan-(1→, →4)-β-D-Glc-(1→, →4)-β-D-Glc-(1→)	*In vivo*	Promoting wound healing	Li et al. (2025b)
LMW-BSP	23.00	Glu: Man = 1.00:1.26	HPGPC, FT-IR, IC, NMR	α-D-Manp-(1 → 3)-β-D-Manp-(1 → [4)-β-D-Glcp-(1]2 → 4)-β-D-Manp-(1 → 3)-β-D-Manp-(1→	*In vivo*	Antitumor activity	Liu et al. (2022a)
BSP	373.00	Man: Glu = 2.946:1	UV, HPGPC, GC–MS, FT-IR, NMR, SEM	(1 → 4)-linked β-D-mannopyranose	*In vivo*	Oral ulcer	Liao et al. (2019)
BSP	198.00	N/A	GPC, FT-IR, NMR	α-D-mannopyranose and β-D-glucopyranose residues	*In vitro*	Anti-osteoarthritis	Lai et al. (2018)
BVPS	147.20	Man: Glu = 26.3:73.7	HPSEC–MALLS, TGA, DSC, XRD, SEM, AFM	N/A	N/A	N/A	Kong et al. (2015)
BFPS	95.45	Man: Glu = 26:74	HPSEC–MALLS, TGA, DSC, XRD, SEM, AFM	N/A	N/A	N/A	Kong et al. (2015)
BSP	139.00	Glu: Man = 2.3:1	HPGPC, GC–MS, TGA, DTA, XRD, FT-IR	N/A	N/A	N/A	Cui et al. (2017)
BSP	236.00	Glu: Man = 1: 3.13	HPGPC, HPLC	N/A	N/A	N/A	Zhou et al. (2022)
BSP	20.00	Glu: Man = 1: 3.5	HPGPC, HPLC, GC-MS, FT-IR, NMR	(1→4)-linked-D-mannose and (1→4)-linked-D-glucose	N/A	N/A	Zhang et al. (2014)
BSP-182	182.10	Glu: Man = 2.2: 7.8	HPLC, FT-IR, GC-MS, NMR, SEM	→3,4)-Glcp-(1→, →3,4)-Manp-(1→, →2,4)-Manp-(1→, →4,6)-Manp-(1→, →4,6)-Glcp- (1→)	N/A	N/A	Ma et al. (2025)
BSP	176.00	Glu: Man = 1: 2.8	HPGPC, FT-IR, NMR	→4)-b-D-Man-(1→4)-β-D-Glc-(1→	N/A	N/A	Zhu et al. (2018)
BSP	116.00	Glu: Man = 1: 2.99	HPGPC, FT-IR, SEM	N/A	N/A	N/A	Qiu et al. (2024b)
BSP	323.70	Glu: Man = 1: 2.4	HPGPC, FT-IR, HPAEC, GC–MS, NMR	→4)-β-Manp-(1→, →4)-β-Glcp-(1→, as well as a small amount of →3)-β-Manp- (1→, terminated by T-Manp residues	N/A	N/A	Hou et al. (2023)

Abbreviations: Man, mannose; Ara, arabinose; Fru, fructose; Fuc, fucose; Glc, glucose; Gal, galactose; Rib, ribose; Xyl, xylose; GluA, gluconic acid; GalA, galactonic acid; HPLC, high-performance liquid chromatography; GC, gas chromatography; FT-IR, Fourier-transform infrared spectroscopy; NMR, nuclear magnetic resonance; GPC, gel permeation chromatography; HPGPC, high-performance gel-permeation chromatography; SEM, scanning electron microscopy; UV, ultraviolet and visible spectrum; GC–MS, gas chromatography–mass spectrometer; HPSEC, high-performance size elusion chromatography; IR, infrared spectroscopy; N/A, not available.

### 4.1 Monosaccharide composition

Monosaccharide composition fundamentally governs polysaccharide bioactivity, necessitating precise characterization as a cornerstone of structural analysis. For BSP profiling, established methodologies include methylation analysis, controlled acid hydrolysis, spectroscopic methods (FT-IR), chromatographic separations (HPLC, GC, and GPC), GC-MS hyphenated systems, and multinuclear NMR spectroscopy. Cumulative evidence confirms BSP fractions are primarily composed of glucose (Glu) and mannose (Man), with variable proportions of galactose (Gal), rhamnose (Rha), arabinose (Ara), and glucuronic acid (GluA). Notably, compositional ratios exhibit significant heterogeneity across studies, attributable to methodological variations in extraction and purification protocols. For example, Qiu M. et al. (2024) demonstrated that BSPs are primarily composed of Glu and Man with a molar ratio of 1:2.99. Chen H. et al. (2023) obtained a homogeneous polysaccharide (BSP-A) from *B. striata* and used a Dionex ICS-3000 linked to a Carbo Pac PA1 Analytic Column (250 × 2 mm) and a CarboPac PA1 Guard Column (50 × 2 mm) to determine that it contained Man, Glu, Ara, Gal, and GalA, with Man and Glu being the predominant monosaccharides. Zhai et al. (2021) isolated four BSPs (BSP-1, BSP-2, BSP-3, and BSP-4) using DEAE-52 cellulose column chromatography. Compositional analysis revealed distinct monosaccharide profiles among the four water-soluble polysaccharide fractions (BSP-1 to BSP-4), with Man and Glu constituting the predominant monosaccharides. Wu et al. (2025) conducted an analysis of BSPs using ion chromatography (IC) with a Thermo Fisher ICS 5000 System and a Dionex Carbo Pac PA10 Column, coupled with an electrochemical detector. The results indicate that the BSP is primarily composed of Man (45.73%), Glu (23.75%), and Fru (15.31%), with minor amounts of Ara (12.56%), Gal (1.57%), and Rha (1.08%). The unexpected presence of Fru in Wu et al. (2025) suggests potential enzymatic degradation during extraction, highlighting how processing conditions can alter monosaccharide profiles. Collectively, the documented heterogeneity in BSP monosaccharide profiles primarily arises from methodological variations in *B. striata* processing and polysaccharide preparation protocols.

### 4.2 Average molecular weights

Molecular weight (MW) fundamentally governs the physicochemical properties and bioactivities of polysaccharides, establishing it as a critical structural parameter. For BSP characterization, three principal chromatographic techniques are employed, including SEC, HPGPC with evaporative light scattering detection (ELSD) or refractive index detection (RID), and SEC with multi-angle laser light scattering (MALLS) and RID. As summarized in Supplementary Table 1, BSP MW ranges from 0.94 kDa to 950.30 kDa across studies—a 1000-fold variation primarily attributable to plant source heterogeneity, extraction methodology differences, and purification protocols (Han et al., 2025; Zhang et al., 2014).

These three analytical platforms (SEC, HPGPC–ELSD, and HPGPC–RID) operate via identical size-exclusion chromatography separation mechanics. Macromolecules exceeding pore dimensions elute rapidly due to steric exclusion, while smaller analytes experience differential retention proportional to hydrodynamic size as they penetrate the porous matrix during column transit. Linear calibration plots generated with pullulan/dextran standards correlate elution volumes directly to molecular weight. For example, the MW of the water-soluble polysaccharide pBSP1 extracted from *B. striata*, measured using SEC-RID on a Sephadex G-100 column with a dextran standard, was determined as ∼255.17 kDa from elution volume (detected via anthrone-sulfuric acid colorimetric assay) (Qiu J. et al., 2024). HPGPC—operating on size-exclusion principles and hyphenated with ELSD or RID has become the predominant method for BSP molecular weight determination owing to its validated analytical reliability and high-throughput capabilities. Cui et al. determined the MW of a natural polysaccharide from *B. striata* by HPGPC-ELSD, and the result showed the MW of the BSP was 139 kDa (Cui et al., 2017). SEC, also termed gel-filtration chromatography, operates through selective size separation while minimizing nonspecific adsorptive interactions between polysaccharides and chromatographic media. Adsorption suppression requires mobile phase optimization with non-volatile buffering agents at controlled ionic strengths. This volatility requirement fundamentally restricts deployment of non-volatile buffer systems in ELSD-compatible mobile phases, establishing HPGPC–RID as the benchmark technique for BSP MW characterization, owing to its unrestricted mobile phase adaptability. The MW values of BSP-1 and BSP-2 were determined to be 83.54 kDa and 12.60 kDa, respectively (Wang Y. et al., 2019), when measured using HPGPC–RID with glucose standards.

The precision of HPGPC measurements is fundamentally constrained by hydrodynamic parameter divergence stemming from the pronounced physicochemical heterogeneity between polysaccharide analytes and reference standards. SEC–MALLS–RID has concomitantly emerged as the benchmark technique for absolute molecular weight determination of polysaccharide polymers, offering uncompromised accuracy, exceptional reproducibility, and reference-standard independence. For example, using high-performance size elusion chromatography (HPSEC)–MALLS–RID, Kong et al. (2015) determined MW of 147.20 kDa for *B. striata* var. polysaccharide (BVPS) and 95.45 kDa for *B. striata* fermentation polysaccharide (BFPS), exhibiting polydispersity indices (MW/Mn) of 12.18 and 7.29, respectively (where Mn denotes number-average molecular weight). Notably, SEC–MALLS–RID eliminates standardization errors caused by conformational differences between polysaccharides and reference polymers (e.g., dextran), establishing it as the gold standard for absolute molecular weight determination.

### 4.3 Chemical structures

Polysaccharide bioactivity is fundamentally governed by three-dimensional architecture, where specific molecular configurations dictate biological functionality. Consequently, systematic elucidation of BSP chemical topologies is essential to establish structure–activity relationships. Integrated analytical platforms, including FT-IR, methylation analysis, periodate oxidation, Smith degradation, GC–MS, and multidimensional NMR, have characterized key structural motifs over decades. However, research disproportionately focuses on compositional parameters (monosaccharide profiles and molecular weights) over higher-order structural features (branching patterns and spatial conformations).

Wang Y. et al. (2019) purified two aqueous-soluble polysaccharide fractions (BSP1 and BSP2) from *B.* tubers. Comprehensive structural elucidation combined FT-IR spectroscopy, linkage analysis via methylation-GC/MS, and complementary ^1^H/^13^C NMR spectroscopy. Structural analysis revealed linear homopolymer domains: BSP1 and BSP2 contain backbone architectures dominated by consecutive β-1,4-linked D-mannosyl residues and β-1,4-linked D-glucosyl residues. Chen H. et al. (2021) utilized HPLC, GC–MS, FT-IR, methylation, and NMR techniques to ascertain the monosaccharide composition and backbones of BSP-H, BSP-B, BSP-A and BSP-U obtained by hot water extraction, boiling water extraction, alkali-assisted extraction, and ultrasonic-assisted extraction methods. The results displayed that BSP-H, BSP-B, BSP-A, and BSP-U possessed identical monosaccharide composition (Glu, Man, and Gal), with molar ratios of 37.89:60.78:1.32, 47.55:51.30:1.16, 43.07:55.91:1.02, and 38.05:61.04:0.92, respectively. NMR spectroscopy revealed that BSP-A uniquely contains α-(1→4)-glucopyranose motifs, whereas β-(1→4)-mannopyranose and β-(1→4)-glucopyranose residues were consistently present in all four polysaccharide fractions. Similarly, Jiang et al. (2023) extracted a polysaccharide demonstrating liver fibrosis alleviation and found that it contained →4)-β-D-Glc*p*-(1 → 4)-β-D-Man*p*-(1 → 4)-β-D-2aceMan*p*-(1 → 4)-β-D-Man*p*-(1 → 4)-β-D-Glc*p*-(1 → 4)-β-D-Man*p*-(1 → 4)-β-D-Man*p*-(1 → 4)-β-D-3ace-Man*p*-(1→. Ma et al. (2025) extracted a novel *B. striata* polysaccharide (BSP-182) and found that it contained →3,4)-Glc*p*-(1→, →3,4)-Man*p*-(1→, →2,4)-Man*p*-(1→, →4,6)-Man*p*-(1→, →4,6)-Glc*p*- (1→). In addition, Chen et al. (2020a) isolated and purified a water-soluble polysaccharide (pFSP) from the originally discarded fibrous roots part of the Chinese traditional herb, *B. striata*. Periodate oxidation, Smith degradation, and FT-IR spectroscopy were performed, and combined with NMR spectroscopy (^1^H, ^13^C, HSQC, HMBC), the refined pFSP was analyzed, and its primary structure was inferred. pFSP consisted of repeating units: (1→4)-linked-α-D-Glc*p*, (1→4)-linked-β-D-Man*p* and (1→3,6)-linked-β-D-Man*p* units, together with the branches of (1→6)-linked-β-D-Gal*p* and terminated with (1→)-linked-β-D-Man*p* residue.

**FIGURE 3:**
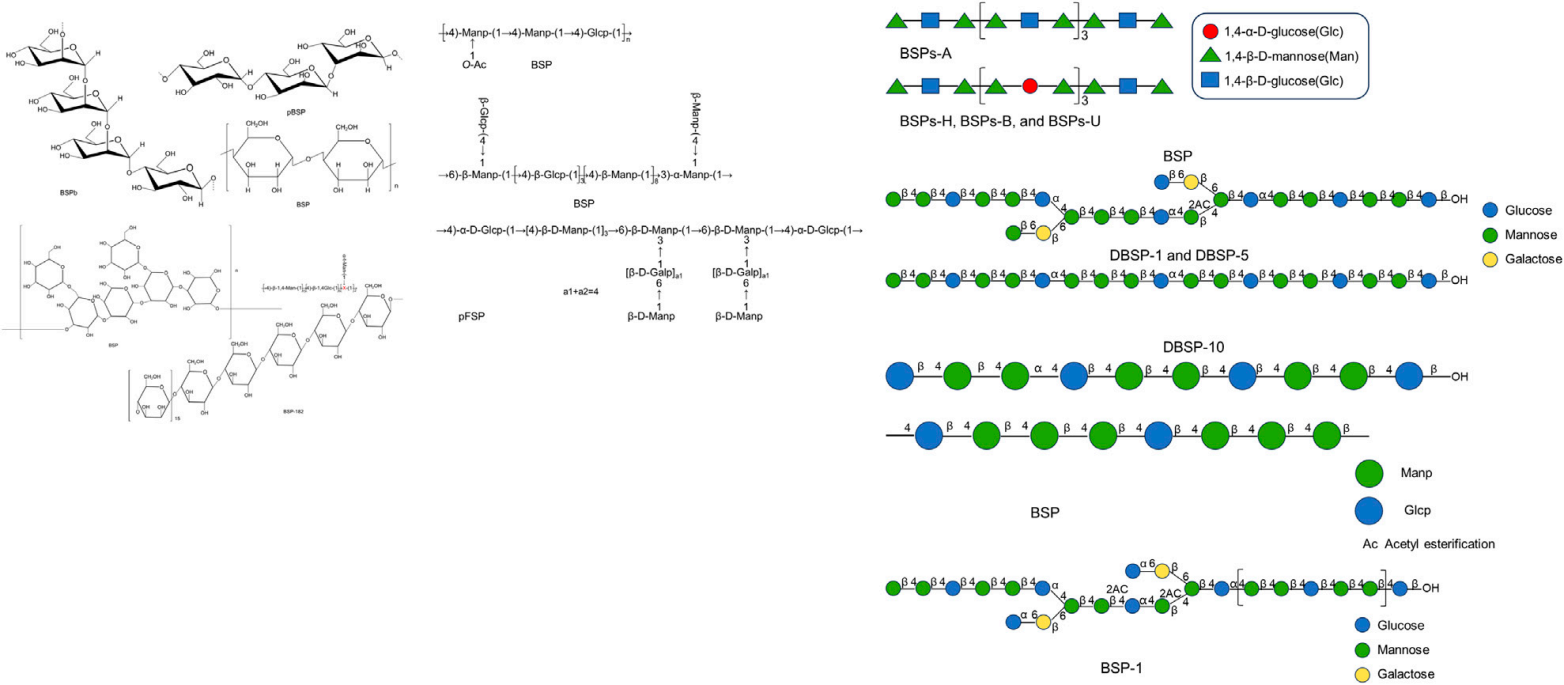
Possible structural features of BSPs (Chen et al., 2024a; Chen et al., 2024b; Chen H. et al., 2021; Chen Z. et al., 2020; Hou et al., 2023; Huang et al., 2024; Ma et al., 2025; Wang et al., 2014; Xu et al., 2021; Zhang et al., 2025; Zhu et al., 2018).

Collectively, an integrated approach (Figure 3) combining chemical derivatization (methylation/Smith degradation) and NMR (HSQC/HMBC) resolves branching patterns.

### 4.4 Conformational features

Polysaccharide biofunctionality is governed by hierarchical structural organization, where nanoscale architecture and spatial conformation dictate biorecognition and bioactivity. These features are resolvable through complementary approaches, including conformation-specific probing (Congo red), nanoscale visualization techniques (SEM and AFM), and crystallographic profiling (XRD). Chen H. et al. (2023) observed irregular fragment morphologies and intricate filamentous networks in BSP-A via field-emission SEM (FE-SEM), suggesting that these coiled filaments originate from triple-helical polysaccharide conformations. The triple-helix structure of BSP-A serves as a fundamental basis for its bioactivity. Although this structural configuration may partially compromise solubility, optimized extraction parameters—including low alkali concentration, moderate temperature, and controlled duration—enable preservation of high extraction yield and favorable solubility profiles. Consequently, BSP-A maintains enhanced bioavailability and demonstrates significant potential for practical applications. This stability was corroborated through Congo red binding assays, which confirmed solution-phase triple-helix topology by demonstrating characteristic bathochromic shifts in λ_max_ proportional to NaOH concentration (0.1–0.4 M). Importantly, the triple-helix conformation demonstrates superior radical-quenching efficacy, with significantly elevated antioxidant activity across standardized assays: DPPH (IC_50_ = 2.498 mg/mL), ABTS (IC_50_ = 3.413 mg/mL), FRAP (44.73 ± 2.88 μmol Fe^2+^/g), and ORAC (47.09 ± 5.68 μmol TE/g). Chen H. et al. (2021) examined polysaccharides isolated through hot water extraction, alkali-assisted extraction, ultrasonic-assisted extraction, and boiling water extraction using SEM. This investigation specifically assessed morphological disparities across these five polysaccharide fractions to establish structure–extraction correlations. Analytical outcomes confirmed differential structural architectures across all five polysaccharide fractions, with bioactivity variations directly attributable to their divergent physicochemical characteristics. Cui et al. (2017) employed XRD to analyze the BSP structure, revealing its amorphous character. AFM provides nanometer-scale resolution with exceptional versatility: minimal environmental constraints, compatibility with hydrated/native-state specimens, and adaptability to diverse biopolymers. AFM uniquely resolves single-molecule tertiary structures under physiologically relevant conditions without crystallization or staining artifacts. Kong et al. (2015) employed AFM to characterize BFPS and BVPS nanostructures. Analysis revealed BFPS forms reticulated networks with uniformly distributed channels, whereas BVPS adopts tightly stacked lamellar assemblies resembling cumulus clouds at equivalent concentrations. This structural divergence likely stems from hydrogen bonding dominance in polysaccharide assembly, where hydroxyl groups mediate strong inter- and intramolecular interactions with adjacent chains and water molecules. To date, systematic studies of BSP conformational energetics and topological dynamics remain lacking. Future priorities include thermodynamic profiling of folding pathways, *in situ* conformational monitoring under physiological conditions; and machine learning-driven structure-bioactivity prediction.

### 4.5 Structural modifications

Given that polysaccharide bioactivities (e.g., antitumor, antioxidant, and anti-inflammatory) are intricately linked to their fine chemical structures, chemical modifications, including selenylation (Zhan et al., 2022), sulfation (Niu et al., 2023), phosphorylation (Xia et al., 2021), acetylation (Yuan et al., 2024), and carboxymethylation (An et al., 2022) serve as pivotal strategies to enhance the efficacy of naturally limited bioactive polysaccharides (Xie et al., 2020). These modifications systematically alter molecular weight, substituent characteristics, branching patterns, and spatial conformations through targeted functional-group introduction. Such structural engineering not only optimizes physicochemical properties but also potentiates bioactivity, with studies confirming that sulfated or selenylated derivatives exhibit significantly enhanced pharmacological profiles compared to native precursors (Fiorito et al., 2018; Otero et al., 2023).

Selenylation represents a particularly promising approach for BSP functionalization, leveraging selenium’s critical role in human metabolism. Studies demonstrate that selenized BSPs have been successfully synthesized using diverse methods, confirming its viability as an active selenized polysaccharide. For example, Jiang et al. (2025) reported that the native BSP exhibits limited *in vitro* antioxidant capacity and minimal *in vivo* attenuation of hepatic fibrosis; therefore, the BSP was chemically modified via the HNO_3_/Na_2_SeO_3_ method to yield the selenized derivative Se-BSP. In brief, for Se-BSP synthesis, 5 g of BSP was dissolved in 500 mL of 0.5% (v/v) HNO_3_ aqueous solution at room temperature. Na_2_SeO_3_ and BaCl_2_ were added, and the mixture was reacted at 70 °C for 6 h under stirring. The reaction mixture was neutralized with NaOH or anhydrous Na_2_CO_3_, and Na_2_SO_4_ was added to precipitate residual Ba^2+^. The slurry was centrifuged at 8000 *g* for 10 min, and the precipitate (BaSO_4_) was discarded. The supernatant was dialyzed exhaustively against deionized water to remove unbound selenium species. Both *in vitro* antioxidant assays and carbon tetrachloride (CCl_4_)-induced murine fibrotic models demonstrated that Se-BSPs significantly enhanced antioxidant activity and attenuated liver fibrosis. Additionally, Se-BSP suppressed α-smooth muscle actin (α-SMA) and collagen I (Col-I) expression, further mitigating liver fibrosis progression. Collectively, these findings establish the selenium-functionalized BSP as a promising candidate for targeted anti-fibrotic therapy. Mechanistically, selenylation introduces -SeO_2_H groups onto glycosyl backbones, potentiating anti-fibrotic activity by enhanced free radical quenching and fibrogenic pathway modulation. This structural modification holds significant promise for targeted anti-fibrotic therapeutics. Initial structure–activity relationship assessments confirm that selenium functionalization amplifies bioactivity, although precise regioselectivity of substitution and molecular targets mediating its anti-fibrotic effects remain uncharacterized. Furthermore, the degree of derivatization and safety profile of introduced selenoglycosidic groups are unknown, highlighting key research priorities. Moreover, the broader pharmacological landscape, including potential enhancement of inherent immunomodulatory/antioxidant activities or the emergence of entirely novel bioactivities, remains unexplored. Equally critical is the deficit in translational studies bridging basic science to preclinical validation and formulation development.

Sulfation stands as one of the most prevalent and efficacious methods for the chemical modification of polysaccharides. The introduction of sulfate groups (-SO_3_H) onto the hydroxyl groups of polysaccharide chains can profoundly alter their physicochemical properties; for instance, it enhances water solubility and chain rigidity, potentially leading to augmented biological activity (Deng et al., 2015; Gong et al., 2023). Notably, research has identified that naturally occurring BSPs contain inherent sulfate groups. Furthermore, the sulfate content in native BSP is variable, and its immunomodulatory potency appears to be correlated with the degree of sulfation (Zhai et al., 2021). This implies that these naturally present sulfate groups are likely critical for mediating BSP’s immune-enhancing effects (Niu et al., 2022). Although no studies have yet reported on the artificial sulfation of BSPs or compared the resultant changes in their immunomodulatory activity, extant evidence strongly infers that modulating BSP’s sulfation level is a highly promising avenue for optimizing its immune-regulatory functions.

## 5 Biological activities of polysaccharides from *B. striata*

**FIGURE 4:**
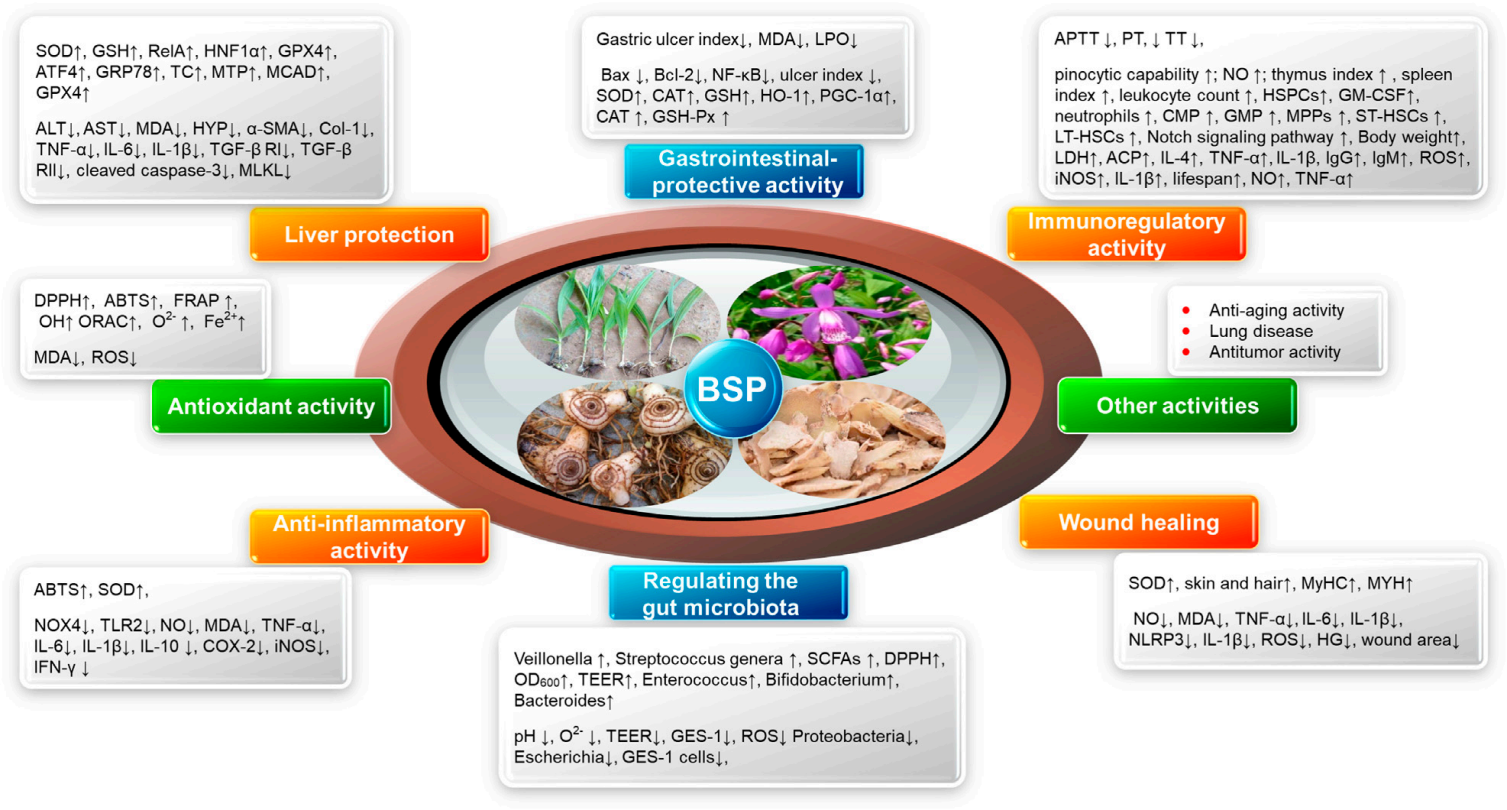
Summary of the biological activities of BSPs.

BSP, the principal bioactive constituents of *Bletilla striata*, exhibit multifaceted therapeutic properties validated through rigorous *in vitro* and *in vivo* studies. This section comprehensively examines current research on BSP’s pharmacological properties, including antioxidant, immunomodulatory, wound healing, gastroprotective, anti-inflammatory, gut microbiota regulation, and hepatoprotective effects. As summarized in Figure 4, these bioactivities position BSPs as promising candidates for nutraceutical and pharmaceutical development.

### 5.1 Antioxidant activity

**FIGURE 5:**
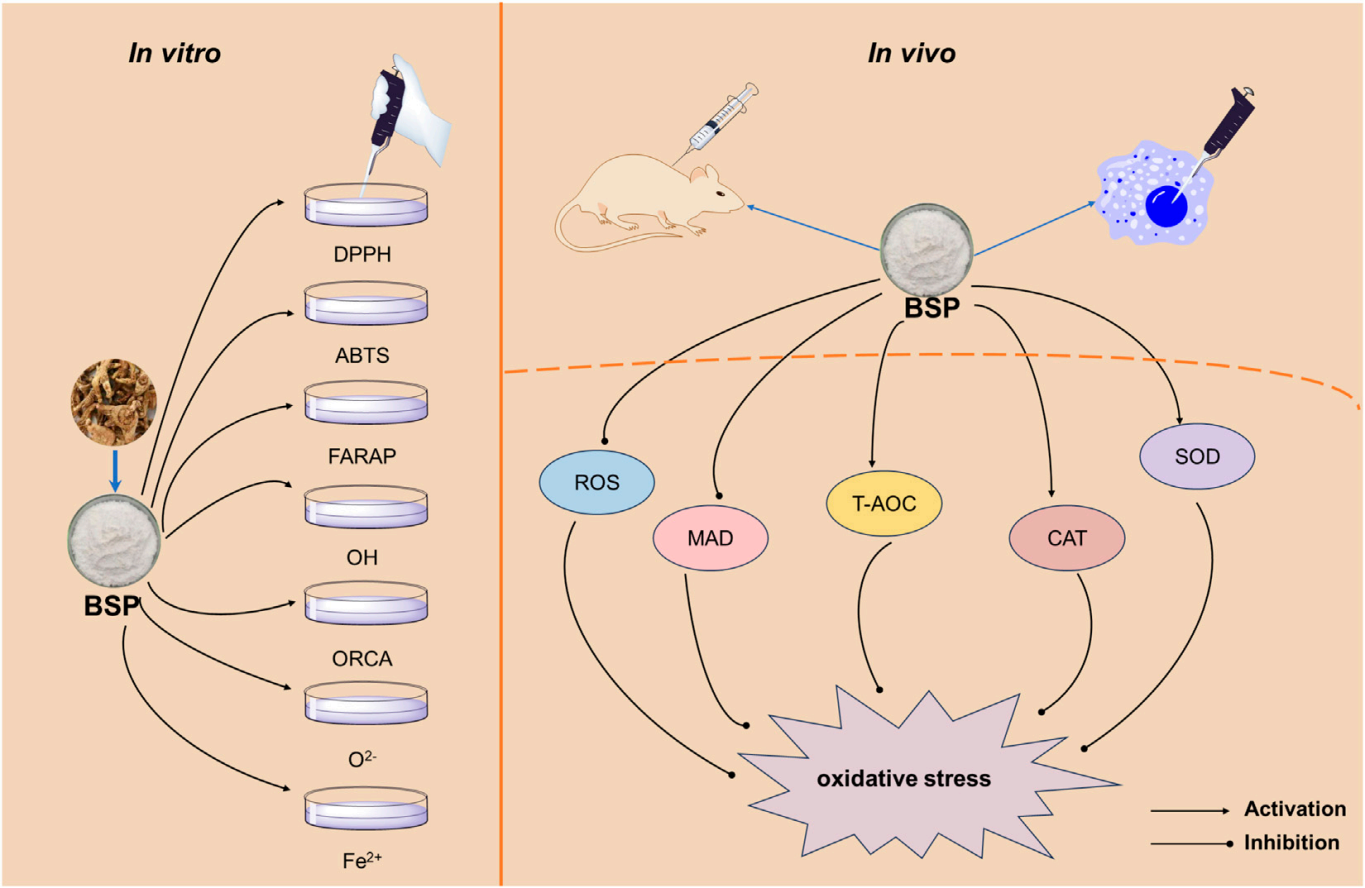
Schematic diagram showing the basic mechanism of antioxidant action of BSPs.

Oxidative stress—characterized by excessive reactive oxygen species (ROS) accumulation—induces a redox imbalance that accelerates cellular senescence and contributes to multiple disease pathologies. This imbalance between oxidant generation and antioxidant defenses triggers neutrophilic inflammation, protease activation, and cytotoxic oxidative damage (Sahragard and Jahanbin, 2017). The mechanisms underlying BSP’s antioxidant activity are illustrated in Figure 5.

Quantitative *in vitro* assessments demonstrate BSP’s dose-dependent radical scavenging capacity. Qu et al. (2016) demonstrated that an infrared-assisted extract of BSPs, at a concentration of 5.0 mg/mL, exhibited scavenging rates of 43.70% against DPPH radicals, 35.97% against hydroxyl radicals, and 42.20% against superoxide anion radicals. The same study noted a ferrous ion (Fe^2+^) chelation capacity of 36.78%, indicating a multi-modal antioxidant action. More recently, Luo et al. (2023) isolated a purified fraction, BSP-2, which showed markedly enhanced activity. At 5 mg/mL, BSP-2 achieved a 73.97% DPPH scavenging rate and a 51.44% ABTS neutralization rate, underscoring the importance of purification in maximizing potency. It significantly outperforms some other polysaccharides, such as the exopolysaccharide EPS2-1, which showed only 20.55% DPPH scavenging at the same concentration (Li Z. et al., 2022). However, compared to other highly active polysaccharides, BSP-2’s activity is moderate to strong but not elite. For instance, a polysaccharide from *Aspergillus wentii* (EPS-AG7) demonstrated 85.90% DPPH and 58.64% ABTS scavenging at 5.0 mg/mL (Ibrahim et al., 2024). At the cellular level, Xu et al. (2021) provided crucial evidence of BSP’s protective effects in a biological context. Using H_2_O_2_-stressed fibroblasts, they found that a purified BSP (pBSP) fraction, at a low concentration range of 0.05–0.20 mg/mL, significantly reduced intracellular ROS production by 32%–61%. This demonstrates that BSPs can effectively counteract oxidative stress within cells, protecting them from damage. Animal studies corroborate the *in vitro* and cellular findings, revealing systemic antioxidant effects. In a subacute aging murine model, He et al. (2021) observed that the BSP restores endogenous antioxidant enzymes (SOD and CAT) and reduces lipid peroxidation (MDA) in aging mice, aligning perfectly with the established effects of potent anti-aging polysaccharides. Across numerous studies, successful polysaccharide treatments in murine aging models consistently report significant increases in SOD activity and decreases in MDA levels (Luo et al., 2011; Qiu et al., 2023). These findings are complemented by research from Zhang et al. (2015), who used the *Caenorhabditis elegans* model to show that BSP’s anti-aging effects are linked to its ability to reduce oxidative stress *in vivo*.

### 5.2 Immunoregulatory activity of BSPs

**FIGURE 6:**
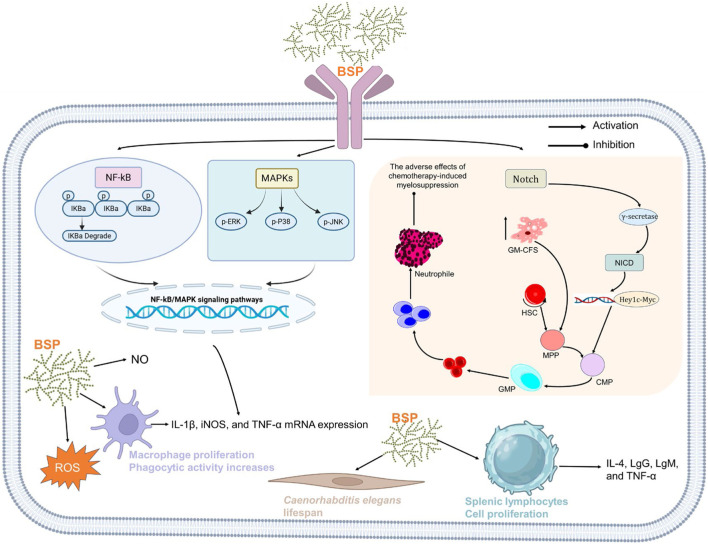
Schematic diagram showing the basic mechanism of the immunomodulatory action of BSPs.

Immunomodulation maintains physiological homeostasis through antigen-specific responses (Alsaffar et al., 2021). As a potent immunostimulant, the BSP exhibits significant immunostimulatory properties through multiple mechanisms (Figure 6).

The foundation of research into BSP’s immunomodulatory effects has been built upon *in vitro* studies, with the murine macrophage cell line RAW264.7 serving as the predominant experimental model. Macrophages are pivotal cells in the innate immune system, responsible for phagocytosis, pathogen recognition, and the orchestration of broader immune responses through the activation of other immune cells. Zhai et al. (2021) demonstrated that purified BSP fragments could enhance the immunomodulatory activity of RAW264.7 cells by increasing their metabolic activity and, significantly, their production of nitric oxide (NO) without negatively affecting cell viability. NO is a key signaling molecule and a primary indicator of macrophage activation and cytotoxic potential. This foundational observation has been corroborated by numerous subsequent reports (Guan et al., 2022; Hu T. G. et al., 2022; Wang Y. et al., 2023). Further investigation by Niu et al. (2022) expanded on these findings, showing that a water-soluble BSP isolate not only enhanced macrophage activity but also increased the production of ROS and elevated the expression of key pro-inflammatory cytokines, including interleukin-1β (IL-1β) and tumor necrosis factor-α (TNF-α). The secretion of these cytokines is a hallmark of macrophage activation and is crucial for initiating and amplifying an inflammatory response to pathogens or cellular stress (Liu and Liu, 2022). Collectively, the *in vitro* data provide robust evidence that the BSP acts as a direct macrophage activator, stimulating the core functions that initiate an innate immune response.

The immunostimulatory effects of BSPs are underpinned by the activation of specific intracellular signaling cascades. The most consistently implicated pathways in macrophage activation are the nuclear factor-kappa B (NF-κB) and mitogen-activated protein kinase (MAPK) signaling pathways. Niu et al. (2022) concluded that BSP-mediated macrophage activation and the subsequent production of inflammatory mediators were primarily mediated through these two pathways. This finding is strongly supported by additional research confirming that NF-κB and MAPK activation are the underlying mechanisms for the immuno-enhancement observed with BSP treatment. These pathways are central hubs for transducing signals from pathogen-recognition receptors, leading to the transcriptional upregulation of genes for pro-inflammatory cytokines, chemokines, and other mediators of the immune response. The findings from Zhang et al. (2025) add another layer of mechanical complexity, identifying the Notch signaling pathway as crucial for BSP’s effects on hematopoiesis and myelopoiesis. This demonstrates that BSP’s immunomodulatory actions are not limited to the direct activation of mature immune cells but extend to influencing the fundamental processes of immune cell development and stem cell proliferation.

The immunostimulatory effects observed in cell culture have been successfully translated into preclinical animal models, confirming BSP’s efficacy in a complex biological system. These studies have primarily utilized mouse models of immunosuppression, often induced by the chemotherapeutic agent cyclophosphamide (CTX), which depletes immune cell populations. In such models, BSPs have demonstrated a potent ability to restore immune homeostasis. Wang Y. et al. (2019) reported that a specific fraction, BSP-1, effectively restored the thymus and spleen indices in immunosuppressed mice. These immune organs are critical for the maturation and function of lymphocytes, and their restoration indicates a recovery of systemic immune capacity. Similarly, Niu et al. (2022) found that BSP administration in CTX-treated mice significantly increased immune organ indices, effectively ameliorated splenic damage, and boosted systemic markers of a humoral immune response, including serum immunoglobulins such as IgG. Furthermore, the study confirmed an increase in serum levels of the cytokines TNF-α and IL-4, underscoring BSP’s ability to modulate immune signaling *in vivo*. Zhang et al. (2025) investigated BSPs in mouse models of chemotherapy-induced myelosuppression, a common and severe side effect of cancer treatment characterized by the depletion of bone marrow and peripheral blood cells. Their findings demonstrated a sophisticated and multifaceted mechanism of action. BSPs were shown to activate the Notch signaling pathway, a critical regulator of cell fate decisions. This activation induced the generation and mobilization of mature myeloid cells, particularly neutrophils, into the peripheral circulation. Concurrently, at the level of the bone marrow, BSP promoted the expansion of hematopoietic stem and progenitor cells (HSPCs) and induced a state of “trained immunity.” This innate immune memory mechanism allows for a more robust response to subsequent challenges.

To contextualize the potential of BSPs, it is useful to compare them with other well-established immunomodulatory polysaccharides, such as beta-glucans (including lentinan), which are derived from fungi, yeast, and cereals (El Khoury et al., 2012; Murphy et al., 2023). Beta-glucans are known to activate immune responses through similar mechanisms, including macrophage activation and cytokine production (Murphy et al., 2023; Stier et al., 2014). However, an extensive review of the available research indicates a significant knowledge gap in this area. There are no published studies that conduct a direct, head-to-head comparison of the immunomodulatory efficacy of *B. striata* polysaccharide and beta-glucan under identical experimental conditions (Vetvicka et al., 1996; Zhai et al., 2021). Therefore, while both classes of polysaccharides are potent immunomodulators, their relative potency remains undetermined. Similarly, there is a lack of direct comparative data on their safety profiles. Although preclinical studies on BSPs have not reported significant toxicity, formal, comparative safety, and toxicology studies are necessary to fully characterize their profile relative to established agents such as beta-glucan.

### 5.3 Anti-inflammatory activity

**FIGURE 7:**
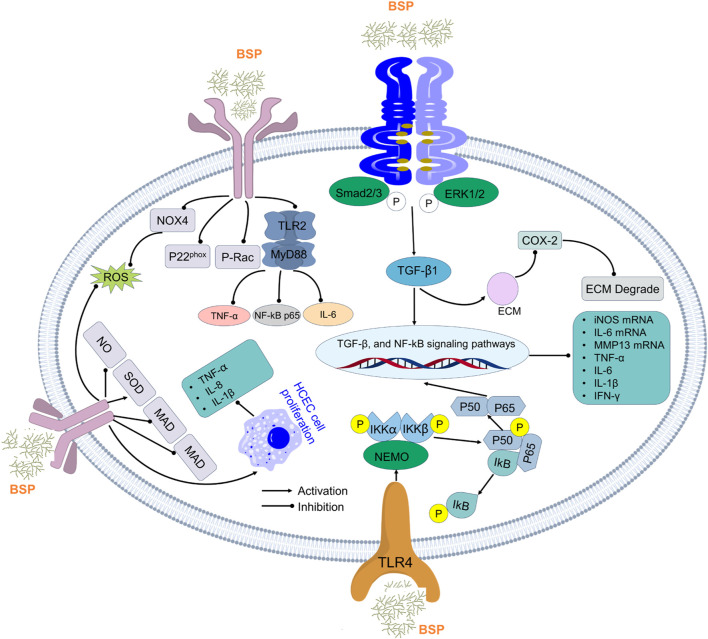
Anti-inflammatory activity mechanism of polysaccharides from *B*. *striata*.

Inflammation represents a critical host defense mechanism against pathogens and injury, yet excessive or chronic inflammation contributes to tissue damage and pathology (Flórez-Fernández et al., 2023; Gurung and Kanneganti, 2016). The clinical management of inflammatory disorders largely relies on conventional anti-inflammatory drugs; however, their utility is often constrained by significant limitations, including adverse side effects and limited efficacy in chronic conditions (Wang B. et al., 2024). Natural polysaccharides such as those from BSPs offer promising alternatives due to their presumed safety, biocompatibility, and, most importantly, their multi-target anti-inflammatory mechanisms (Figure 7). Emerging research indicates that BSPs demonstrate significant anti-inflammatory effects through profound immunomodulation, characterized by the suppression of core inflammatory signaling pathways, a marked reduction in pro-inflammatory cytokine production, and the potentiation of anti-inflammatory mediators. These mechanisms appear to act synergistically to attenuate pathological inflammation and help maintain immune homeostasis, with proposed underlying actions involving the direct modulation of immune cell function and the enhancement of endogenous antioxidant defenses. For example, Guo et al. (2020) reported a specific polysaccharide from *B.*, designated BSPS, which elicited potent anti-inflammatory activity by decreasing pro-inflammatory mediators (TXB2, TNF-α, IL-6, and IL-1β) while simultaneously increasing anti-inflammatory cAMP and IL-10 levels *in vivo*. This dual action of suppression and promotion is a hallmark of sophisticated immunomodulation. Furthermore, early *in vitro* work showed that BSPs demonstrated significant anti-inflammatory activity with inhibitory effects on the expression of IL-1β, TNF-α, and inducible nitric oxide synthase (iNOS) in the murine macrophage cell line RAW264.7 at a concentration of 200 μg/mL (Diao et al., 2008). More recent investigations sought to elucidate the properties of specific BSP fractions; researchers isolated two distinct fractions, BFP60 and BFP80, and subsequent bioactivity assays demonstrated potent anti-inflammatory effects for both. Mechanistic studies revealed that both BFP60 and BFP80 significantly suppressed nitric oxide (NO) production and the secretion of key pro-inflammatory cytokines (IL-6, IL-1β, TNF-α, and IFN-γ) (Gu et al., 2022). Their protective effects against inflammation are believed to occur primarily through the inhibition of NF-κB pathway activation and the subsequent downregulation of iNOS and cyclooxygenase-2 (COX-2) protein expression, two critical enzymes in the inflammatory cascade.

The compelling anti-inflammatory properties of the BSP are not limited to a single mode of action but rather a coordinated suppression of multiple inflammatory processes. This multi-pronged approach distinguishes it as a particularly robust immunomodulatory agent. A significant body of research confirms that the BSP exerts its effects by targeting central nodes of the inflammatory response. The NF-κB pathway, a master regulator of inflammatory gene expression, is a primary target. As demonstrated by Gu et al. (2022), BSP fractions directly inhibit NF-κB activation, thereby preventing the transcription of numerous downstream targets, including cytokines and enzymes like COX-2. In addition to NF-κB, the BSP demonstrates a remarkable ability to inhibit the NLRP3 inflammasome, a multi-protein complex critical for processing and activating the potent pro-inflammatory cytokines IL-1β and IL-18 (Zhao et al., 2021). Studies show that the BSP effectively promotes diabetic wound healing by inhibiting NLRP3 inflammasome activation and subsequent IL-1β secretion in macrophages (Ji et al., 2020). This specific targeting of the inflammasome is a sophisticated mechanism that is not universally reported for all anti-inflammatory polysaccharides and represents a key advantage as dysregulated NLRP3 activity is implicated in a wide range of chronic inflammatory and metabolic diseases. The downstream effect of BSP’s pathway inhibition is a broad-spectrum reduction in the production of pro-inflammatory molecules. Across numerous *in vitro* and *in vivo* models, BSP and its derivatives have been shown to significantly reduce levels of hallmark inflammatory cytokines, including TNF-α, IL-6, and IL-1β (Zhu et al., 2022; Zu et al., 2019). This effect has been observed in various contexts, from protecting cells against LPS-induced injury to mitigating inflammation in murine models of ulcerative colitis and acute lung injury.

The potent biological activities of polysaccharides are intrinsically linked to their structural characteristics, including molecular weight, monosaccharide composition, and glycosidic linkages (Zhang Y. et al., 2024). BSP is primarily a glucomannan, composed of D-glucose and D-mannose residues, forming a backbone of β-1,4-linked mannosyl and glucosyl units (Ji et al., 2020). Research suggests that specific structural features, such as lower molecular weights, and certain glycosidic linkages, such as β-(1→3) and β-(1→6), can correlate with enhanced anti-inflammatory activity in polysaccharides generally (Zhang Y. et al., 2024). Although the precise structure–activity relationship for BSP is still an area of active investigation, it is highly plausible that its unique glucomannan structure, with its specific branching patterns and acetyl group substitutions, creates a three-dimensional conformation that is uniquely recognized by immune cell receptors, leading to its powerful modulatory effects.

### 5.4 Wound healing

BSPs exhibit significant wound-healing bioactivity, mediated primarily through activation of the TGF-β/Smad signaling pathway (He X. et al., 2024). At the cellular level, low concentrations (5–10 μg/mL) of the BSP enhance proliferation and migration in fibroblasts (L929) while potently activating myocytes (C2C12), augmenting their proliferation (+10%), differentiation (+1.5–4 fold), migration (+15–70%), and invasion capability (+1.84–4.65-fold). In translational applications, BSP-incorporated hydrogel (40 mg/mL) accelerates full-thickness wound repair by maintaining a moist wound microenvironment and achieving complete closure within 12 days (versus unhealed controls). Histological analyses confirm that the BSP significantly enhances epithelial regeneration and collagen deposition. This bioactivity stems from multi-target mechanisms: significant suppression of key pro-inflammatory factors (TNF-α, IL-1β, IL-6, and iNOS) synthesis/release, enhanced antioxidant capacity (*P* < 0.05), coupled with anti-inflammatory, antioxidant, and analgesic effects (He X. et al., 2024). Critically, in diabetic pathologies (Zhao et al., 2021), the BSP accelerates refractory wound repair by suppressing hyperglycemia-induced NLRP3 inflammasome activation (TXNIP/NLRP3/caspase-1 pathway), reducing macrophage-derived IL-1β secretion, and improving endothelial insulin sensitivity. Notably, fresh-tuber-derived BSPs achieve epithelialization and wound closure efficacy comparable to recombinant bovine basic fibroblast growth factor (rb-bFGF) (He Z. et al., 2024).

### 5.5 Gastrointestinal-protective activity

Although gastric secretion of proteolytic enzymes and HCl is essential for digestion (Haniadka et al., 2013), excessive aggression can cause mucosal injury. BSPs counteract this damage through multi-target protection mechanisms (He et al., 2017).

A primary driver of mucosal damage following insults, such as ethanol exposure, is the massive generation of ROS, which leads to lipid peroxidation (LPO), protein damage, and, ultimately, cellular apoptosis. BSP is a potent regulator of these interconnected pathways. Wang et al. (2020) demonstrated this dual capacity in both *in vitro* and *in vivo* models of ethanol-induced gastric injury. *In vivo*, oral administration of BSPs led to a significant suppression of the ulcer index. Mechanistically, the BSP significantly enhances the activities of superoxide dismutase (SOD), catalase (CAT), and glutathione peroxidase (GSH-Px), reduces the levels of malondialdehyde (MDA) and lipid hydroperoxide (LOOH), directly clears ROS, and repairs oxidative damage. However, existing research on polysaccharides (such as seaweed polysaccharide Cm-SP) mainly exerts protective effects through prostaglandin (PG)-dependent pathways, and high doses (200 mg/kg) are required to achieve a 92% gastric injury inhibition rate (Carneiro et al., 2018). Wang et al. (2017) revealed that the BSP modulates the expression of key genes controlling programmed cell death. It upregulated the anti-apoptotic gene Bcl-2 while downregulating the pro-apoptotic gene Bax, thereby shifting the cellular balance away from death and toward survival. This anti-apoptotic effect was further confirmed by Qiu J. et al. (2024), who found that a purified BSP fraction (pBSP1) significantly attenuated ROS generation and apoptosis in LPS-induced gastric epithelial (GES-1) cells. Their study revealed that pBSP1 at 400 μg/mL ameliorated LPS-induced cellular damage by suppressing transepithelial electrical resistance (TEER) reductions in Caco-2 cells and attenuating apoptosis and ROS generation in both cell lines, thereby exerting gastroprotection.

### 5.6 Liver protection

**FIGURE 8:**
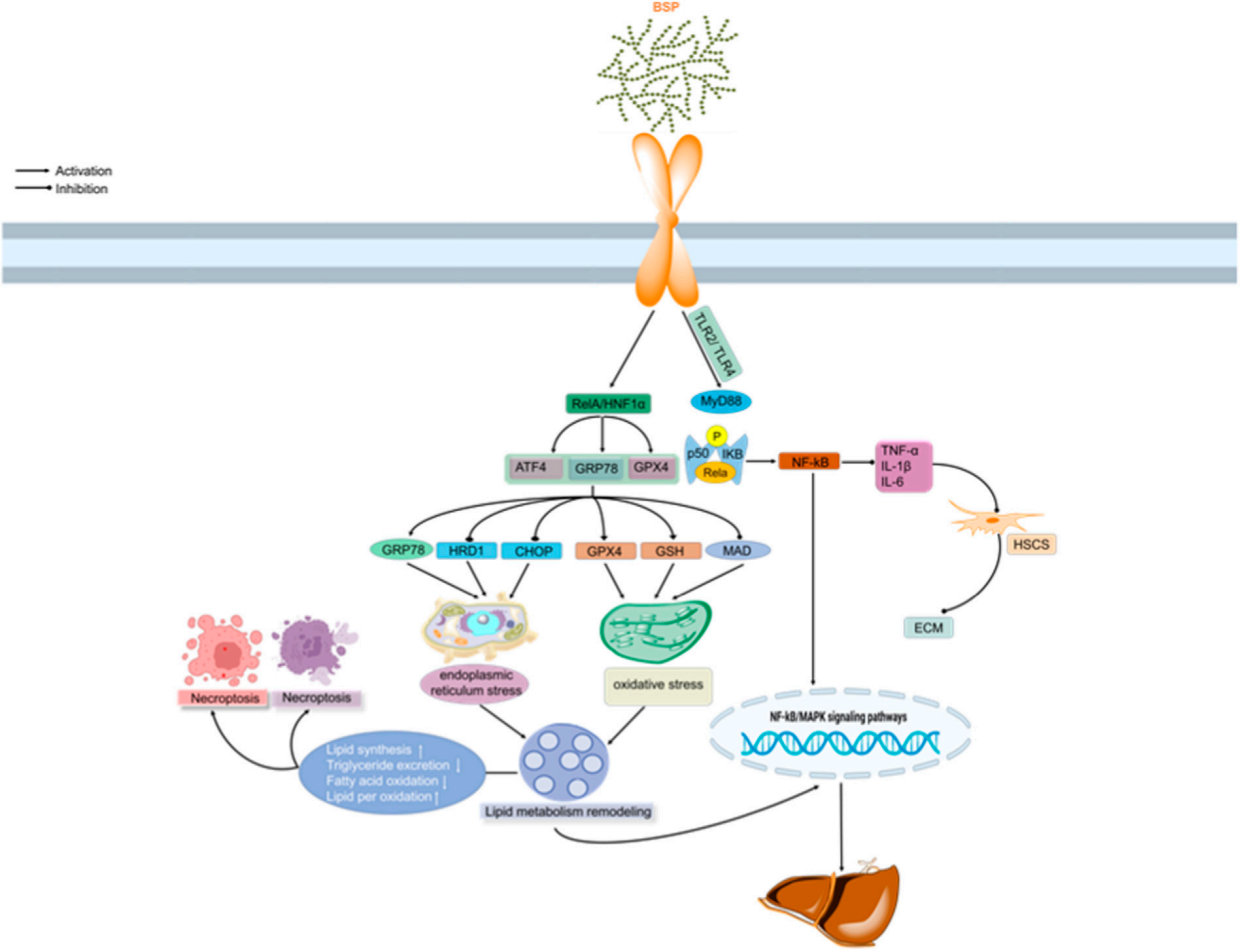
Possible mechanisms of hepatoprotective effects of BSPs.

As the primary organ for systemic metabolic regulation and detoxification (Trefts et al., 2017), the liver is vulnerable to injury from multiple hepatotoxic factors, including pharmaceuticals, industrial chemicals, dyslipidemia, and chronic ethanol intake, which promote hepatocellular damage and hepatic pathogenesis (Yi Hui Toy et al., 2022). Compared to synthetic hepatoprotectants, BSPs offer superior biocompatibility and reduced adverse effects, with distinct therapeutic advantages including potent antioxidant capacity, mitochondrial protection in hepatocytes, and metabolic regulation. In a murine non-alcoholic steatohepatitis model, Hu et al. (2022a) demonstrated that BSP intervention significantly attenuated pathological features, including reduced body weight in high-fat diet (HFD)-fed mice, suppressed ALT elevation, and lowered serum TC, TG, and LDL-C levels. Metabolomic profiling further revealed that these improvements were mediated through the suppression of fatty acid metabolism regulators (oleic acid, docosahexaenoic acid, α-linolenic acid, and γ-linolenic acid) and critical metabolic intermediates (uric acid, D-3-phosphoglyceric acid, cytidine, and 6-aminocaproic acid), thereby ameliorating systemic lipid metabolic dysregulation. These effects collectively ameliorated systemic lipid dysregulation, with possible mechanisms illustrated in Figure 8.

### 5.7 Regulating the gut microbiota

Thr BSP functions as a prebiotic modulator of gut microbiota—a critical determinant of host homeostasis and systemic health. Through selective fermentation, BSPs enrich beneficial taxa and generates bioactive metabolites, as evidenced by short-chain fatty acid (SCFA) biosynthesis enhancement. *In vitro* fermentation studies revealed the intestinal modulatory properties of *B. striata*-derived polysaccharides BP and BO. Additionally, post-fermentation broths exhibited potent antioxidant activity: BO broth reached 68.27% ± 0.27% DPPH radical inhibition at 12 h, whereas BP broth showed significantly greater DPPH inhibition than controls only after 24 h (*p* < 0.01). Strikingly, BP broth showed significantly enhanced superoxide anion (O_2_^−^) scavenging versus BO and controls (*p* < 0.01), with the latter groups displaying minimal O_2_^−^ neutralization. Collectively, results confirm BP/BO promote SCFA generation via selective microbiota reprogramming, thereby mediating distinct antioxidant responses that underscore their promise as prebiotic candidates for therapeutic applications (Wang Q. et al., 2023). Furthermore, the distinct SCFA profiles induced by BP and BO suggest their differential activation of host signaling pathways. Butyrate, predominantly produced from BP fermentation, is known to enhance intestinal barrier integrity and serve as an HDAC inhibitor, potentially contributing to anti-inflammatory effects. In contrast, propionate from BO may play a more significant role in modulating glucose and lipid metabolism via hepatic signaling. These findings highlight the structure-dependent prebiotic mechanisms of BP and BO, underscoring their potential tailored applications in metabolic and inflammatory disorders. Collectively, BSP-mediated microbiota reprogramming simultaneously enhances SCFA generation and confers distinct antioxidant activities, supporting its therapeutic application for managing microbial dysbiosis.

### 5.8 Other biological activities

#### 5.8.1 Anti-aging activity

Although currently reported in a single study, BSPs demonstrate significant anti-aging potential in *Caenorhabditis elegans* models (Zhang et al., 2015). At 50 μg/mL, BSPs extended lifespan by 20.3%, enhancing locomotion ability (body bends and head thrashes) and improving stress resistance to thermal/oxidative challenges. Mechanistic studies revealed that BSPs downregulate *age-1* and *hcf-1* genes in the insulin/IGF-1 signaling pathway, with lifespan extension fully dependent on *daf-16*. Critically, BSPs exerted no antibacterial effects or reproductive toxicity, confirming specific geroprotective activity. This establishes the BSP as a novel therapeutic candidate for aging intervention.

#### 5.8.2 Chronic obstructive pulmonary disease

Li L. et al. (2024) demonstrated BSP’s therapeutic efficacy against chronic obstructive pulmonary disease (COPD) through a novel gut–lung axis mechanism. In both cigarette smoke extract (CSE)-induced human bronchial epithelial cells and murine COPD models, BSP treatment activated the NR1H4/FXR pathway via gut microbiota-mediated enrichment of *Bacteroides intestinalis*, significantly reduced pulmonary cytokine levels (IL-6, TNF-α, and IL-1β; *p* < 0.01 vs. COPD control), and improved lung function parameters. This study establishes the gut microbiota-NR1H4/FXR pathway as a critical therapeutic target for COPD, with BSP serving as a potent modulator of this axis.

#### 5.8.3 Antitumor activity

Natural polysaccharides demonstrate potent anticancer activity through immunomodulation, apoptosis induction, and metastasis suppression (Jin et al., 2025; Lu et al., 2023). Specifically, low molecular weight BSP (LMW-BSP) exhibits enhanced immunostimulatory effects in tumor microenvironments. Liu C. et al. (2022) demonstrated that in cyclophosphamide-treated H22 tumor-bearing mice, LMW-BSP enhanced immune responses by dose-dependently increasing activity of NK cells, macrophages, lymphocytes, and the CD4^+^/CD8^+^ ratio in peripheral blood T lymphocytes.

## 6 Structure–activity relationships of *B. striata* polysaccharide

**FIGURE 9:**
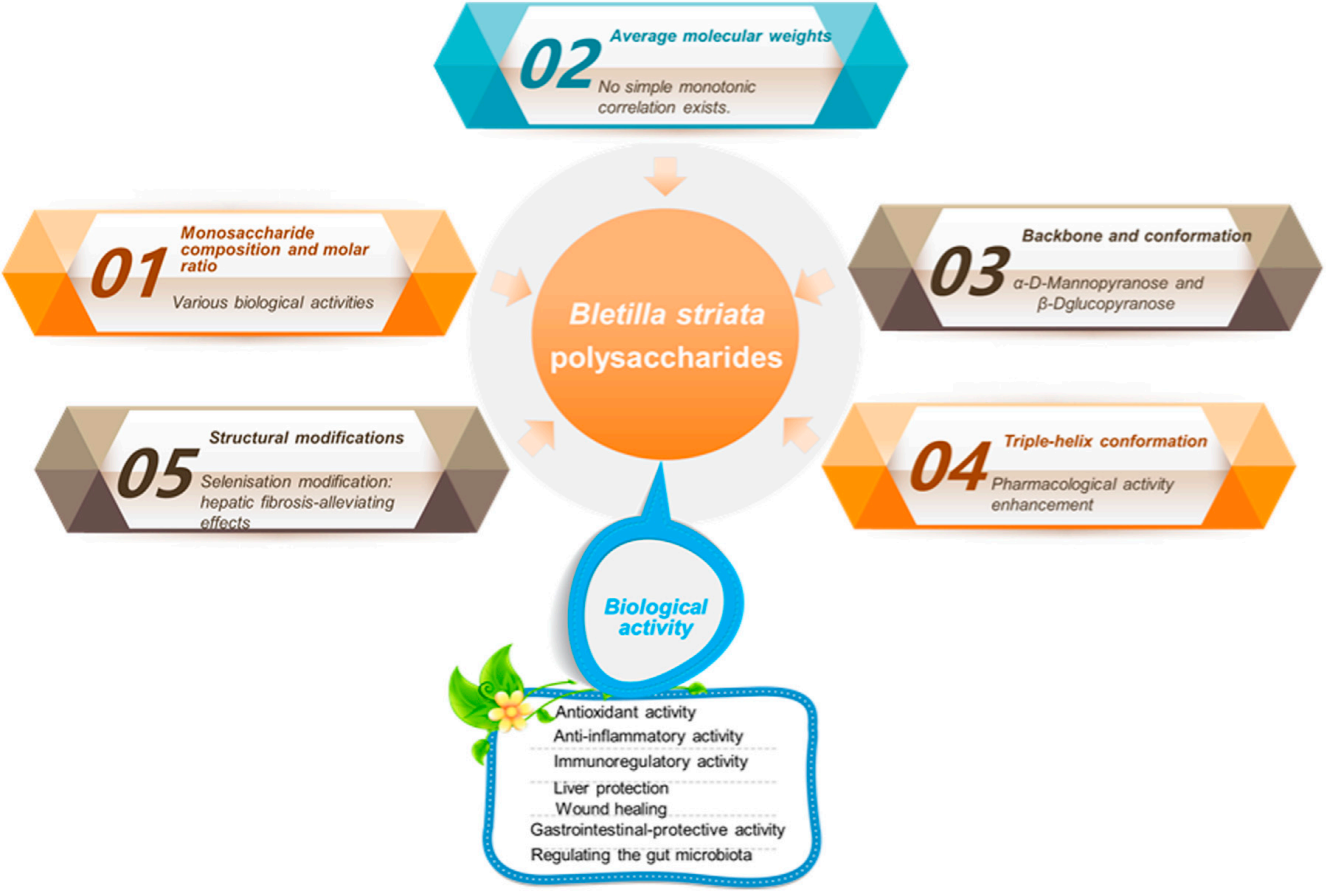
Structure–activity relationship of BSPs.

It is increasingly recognized that the diverse bioactivities of BSP are not monolithic but are intrinsically linked to its complex and heterogeneous molecular architecture. Key structural parameters, including monosaccharide composition, MW, glycosidic linkage patterns, and branching degree, are critical determinants of its functional mechanisms (Figure 9).

**FIGURE 10:**
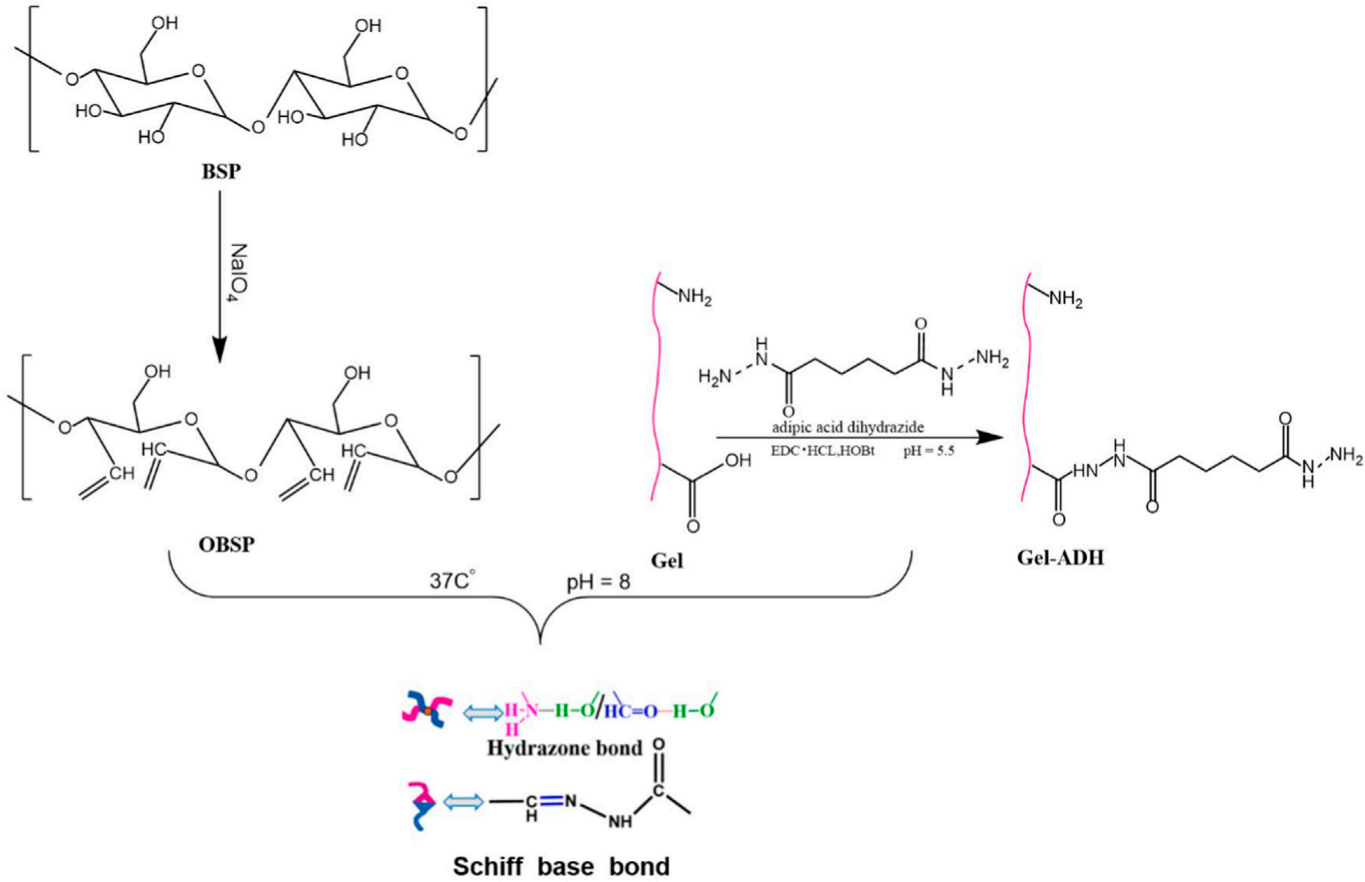
Schematic diagram of OBGTP hydrogel formation process diagram.

**FIGURE 11:**
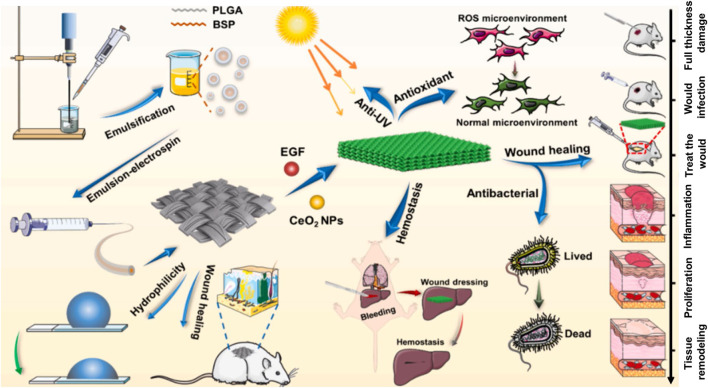
Schematic diagram of EGF@BSP-CeO2/PLGA nanofibrous scaffolds for wound-healing drug delivery.

**FIGURE 12:**
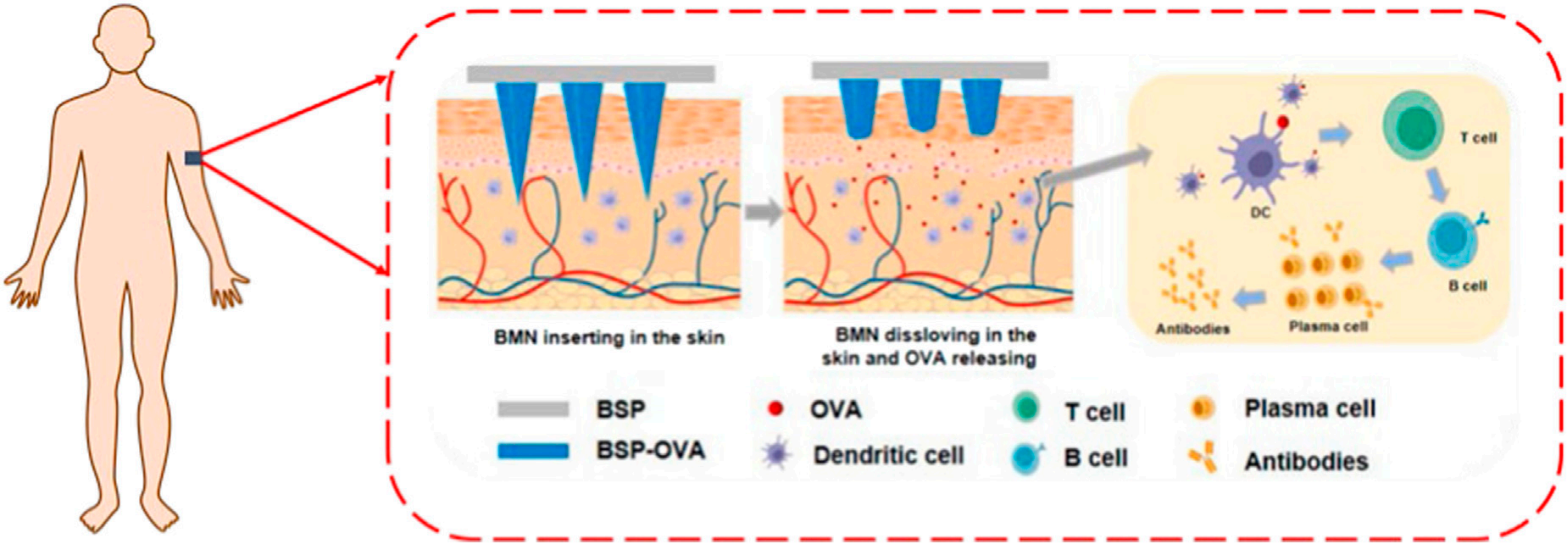
Schematic diagram of transdermal drug delivery of the OVA-loaded BSP microneedle system.

**FIGURE 13:**
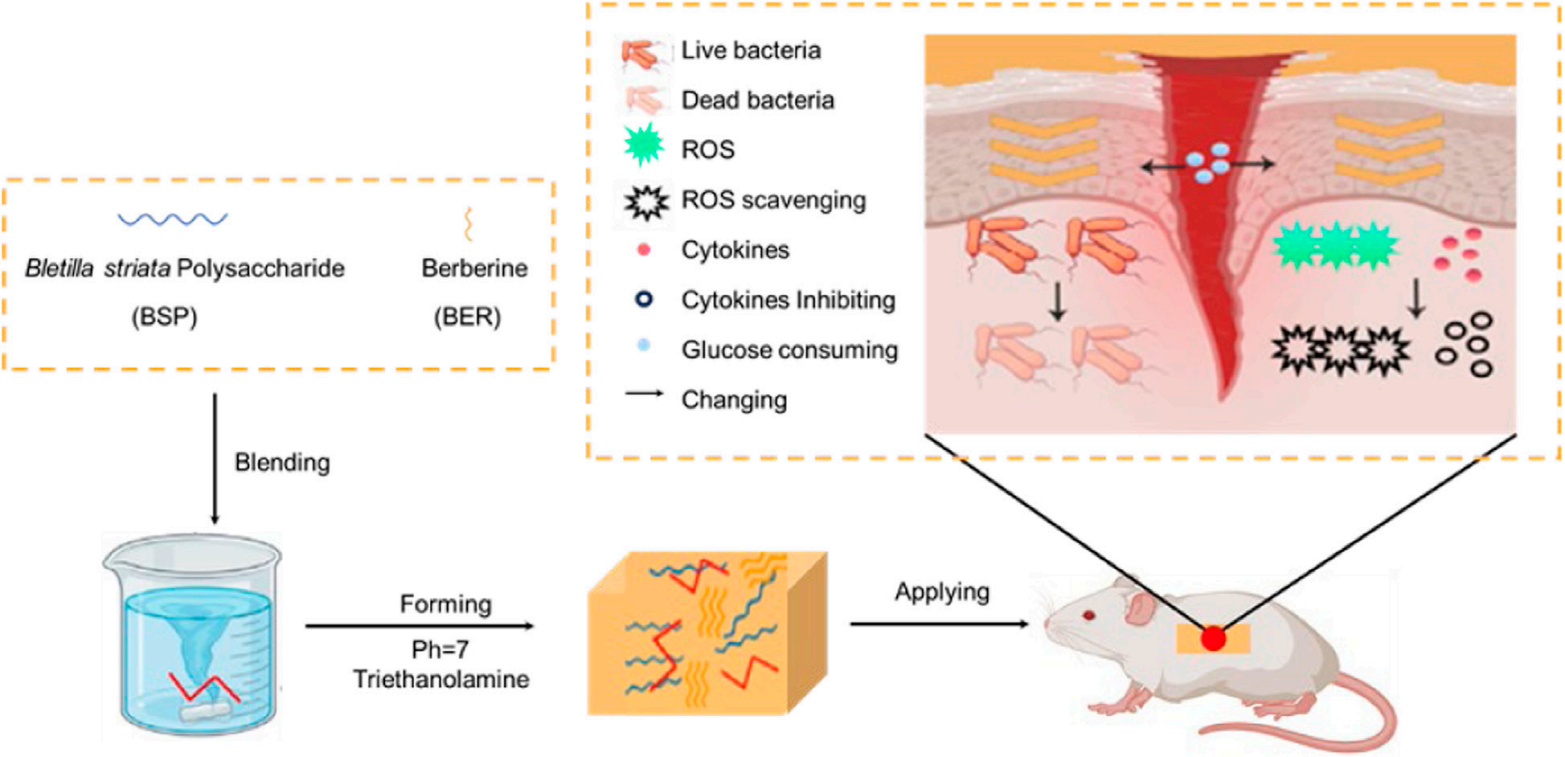
Schematic illustration of the preparation of BSP/BER hydrogel and their mechanism for promoting diabetic wound healing.

**FIGURE 14:**
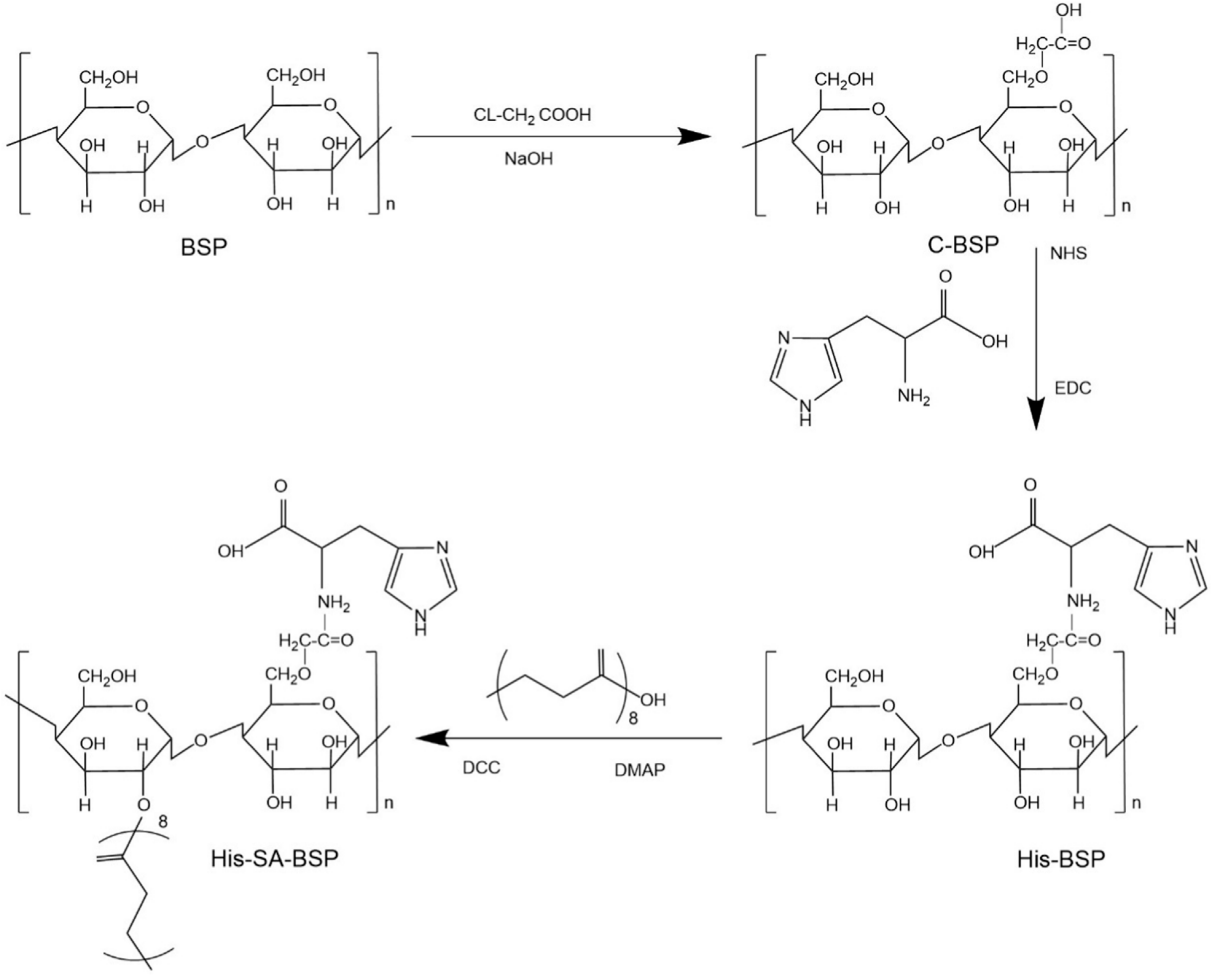
Synthesis scheme of His-SA-BSP.

**FIGURE 15:**
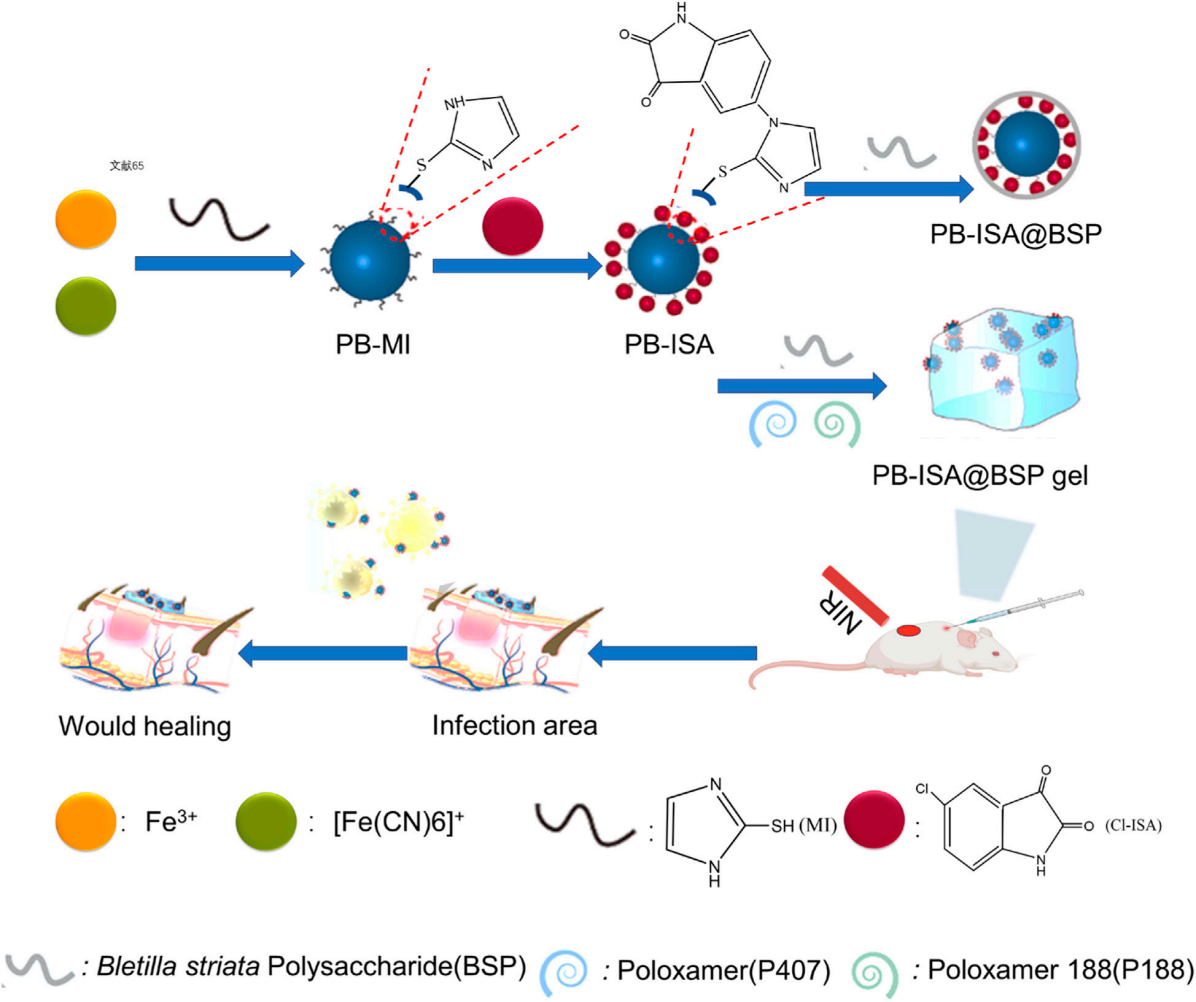
Synthesis scheme of PB-ISA/BSP gel and photothermal ablation mechanism of PB-ISA/BSP gel upon 808 nm laser irradiation.

For instance, BSPs, which contain Man and Glc, exhibits gastroprotective activity (Wang et al., 2020). In contrast, BSPs, consisting of Man, Glc, and Gal, show protection against UVB-induced oxidative stress in skin (Chen et al., 2024a). Even when the monosaccharide composition is identical, the pharmacological effects may still diverge due to variations in the ratios of these monosaccharides. For example, BSP-1 and BSP-2 both have Man: Glu in ratios of 4.0:1.0 and 3.0:1.0, respectively (Wang Y. et al., 2019). BSP-1 exhibited immunomodulatory activity by increasing the thymus and spleen indices in *in vivo* experiments. Moreover, the pharmacological effects of *B. striata* polysaccharides depend on their MWs. For example, the molecular weight (MW) of BP was determined to be 221.17 kDa, whereas the MW of BO, after hydrolysis with trifluoroacetic acid, was 0.94 kDa. A study simulating gut microbiota digestion and fermentation revealed that BP demonstrated stronger SCFA production and antioxidant activity within the gut microbiota compared to BO (Wang Q. et al., 2023).

It is well-established that polysaccharides with either excessively high or low MW often fail to exhibit their biological functions effectively. High-MW polysaccharides exhibit poor cellular uptake due to limited membrane permeability, while low-MW polysaccharides may successfully bind to target sites but frequently lack the tertiary structural complexity necessary for eliciting a potent biological response (Wang et al., 2023d). Since MW is a modifiable structural parameter, further investigation into *B. striata* polysaccharides across a spectrum of MW values will be pivotal for identifying the optimal range that confers robust biological activity, thereby greatly advancing our understanding of its structure–activity relationship. Intriguingly, *B. striata* polysaccharides can elicit comparable pharmacological effects even when their MW and monosaccharide composition differ. For example, BSP, DBSP-1, DBSP-5, and DBSP-10 demonstrate antioxidant activities, even though their MWs differ substantially, at 298.82, 153.94, 66.96, and 15.54 kDa, respectively. BSPs are composed of Glu, Man, and Gal, while DBSP-1, DBSP-5, and DBSP-10 consist of Man and Glu. These similar pharmacological effects may be attributed to the presence of shared structural motifs, such as α-D-Glcp-(1→, →4)-2-O-acetyl-β-D-Manp-(1→, →4)-β-D-Manp-(1→ (Chen et al., 2024a).

Polysaccharides with a regular triple helix conformation typically exhibit significantly higher biological activity than similar polysaccharides with a random coil conformation (Tang et al., 2022). For example, lentinan, a polysaccharide derived from shiitake mushrooms, is thought to exhibit potent anti-tumor activity, which is closely associated with its stable triple-helix conformation; disruption of this structure into a single chain results in a substantial loss of activity (Liu P. et al., 2018). Therefore, establishing whether a target polysaccharide adopts a triple-helix conformation is a crucial prerequisite for elucidating its structure–activity relationship and accurately assessing its bioactivity. Using FT-IR, SEM, and Congo red assays, Chen et al. (2023a) identified a triple-helix-structured polysaccharide, designated BSP-A. It was further demonstrated that BSP-A exhibits notable antioxidant properties.

Chemical modification introduces new functional groups that can profoundly alter or enhance the pharmacological properties of polysaccharides; such targeted structural alterations represent a key strategy for improving their bioavailability and target specificity. Wu et al. (2019) demonstrated that acetylated polysaccharides derived from white adipose tissue can modulate macrophage activation and enhance wound-healing processes. This finding implies that acetylation modifies how BSP interacts with immune cells. The immunomodulatory mechanism of acetylated polysaccharides likely involves their binding to specific cell surface receptors, thereby activating downstream signaling pathways (Liu X. et al., 2018). Although research on the signaling pathways of acetylated BSPs is still insufficient, clues can be gleaned from studies of its parent BSP and other polysaccharides. Studies have shown that native BSP itself can exert immunoenhancing and anti-inflammatory effects by regulating key signaling pathways such as MAPK and NF-κB (Niu et al., 2022). Meanwhile, other plant polysaccharides have also been confirmed to regulate macrophage function by activating AKT/NF-κB, MAPK, and TLR4-mediated signaling pathways (Guo et al., 2022; Qiao et al., 2022; Schepetkin et al., 2013). Therefore, acetylation modification enhances the affinity of BSPs for macrophage surface receptors (e.g., TLR4 on RAW 264.7 cells) by altering its conformation or charge distribution, thereby more effectively activating signaling pathways such as NF-κB and MAPK. This leads to changes in the expression levels of immune-related factors such as NO, TNF-α, and IL-1β, ultimately resulting in stronger immunomodulatory effects, which represents a future research direction.

## 7 Safety assessment

To date, growing scientific recognition of traditional Chinese medicine and heightened health consciousness have driven intensified research on plant-derived bioactives. Polysaccharides as macromolecules exhibiting multivalent biological activities require rigorous safety assessment before therapeutic application. This requires comprehensive toxicological profiling to establish biocompatibility and structure–safety relationships.

### 7.1 *In vitro* safety evaluation

Consistent evidence demonstrates BSP’s low cytotoxicity across multiple cell types. Li L. et al. (2024) conducted cytotoxicity assays on BSPs, demonstrating that concentrations of 5, 10, and 20 μg/mL exhibited no significant reduction in cell viability after 48 h compared to untreated controls. Consequently, subsequent experiments utilized the highest safe concentration of 20 μg/mL. Complementary studies on hot-water-extracted BSPs confirmed this safety profile: MTT assays on normal L929 cells showed no cytotoxicity at concentrations up to 0.32 mg/mL after 24 h of exposure (Wang S. et al., 2024). Further corroborating these findings, Chen et al. (2020b) demonstrated via the MTT assay that BSP concentrations spanning 1–1,000 μg/mL induced no significant cytotoxicity in L929 fibroblasts. All treatment groups maintained over 70% cell viability relative to controls following both 24 h and 96 h exposures. Complementary evidence from Huang et al. (2024) established concentration-dependent immunostimulatory effects in RAW264.7 macrophages: BSPs exhibited no toxicity at 25–200 μg/mL while dose-dependently enhancing proliferation compared to untreated cells (*p* < 0.05). Consequently, concentrations of 25–200 μg/mL were selected for subsequent immunomodulatory studies. He Z. et al. (2024) identified a biphasic response in C2C12 cell myoblasts treated with BSPs across an extended range (3.125–4,000 μg/mL). Notably, BSPs significantly enhanced viability at sub-therapeutic concentrations (3.125–12.5 μg/mL; *p* < 0.001) but exerted cytotoxicity at supra-therapeutic concentrations (200–400 μg/mL; *p* < 0.001), revealing a narrow therapeutic window for myogenic applications.

### 7.2 *In vivo* biosafety evaluation

Complementary evidence confirms biosafety across administration routes. Wu et al. (2024) assessed the *in vivo* biocompatibility of BSPs. Healthy mice received nebulized BSPs (10, 15, and 25 mg/mL) for three consecutive days. Results showed no significant differences in body weight, blood leukocyte/platelet counts, and BALF neutrophil counts, or serum/BALF cytokine levels between BSP-treated and control groups. These collective findings demonstrate the inhalation biocompatibility of BSPs in murine models under the described dosing regimen. To establish systemic biocompatibility, wild-type murine models were employed for toxicological assessment of BSPs. Critically, BSP-treated cohorts (0.1, 0.2, and 0.4 g/kg) exhibited no statistically significant changes in hepatic weight, serum biomarkers (ALT, TC, and TG) (*p* > 0.05) and showed no significant difference in hepatic TG accumulation versus controls, collectively confirming BSP’s metabolic safety and minimal hepatotoxic potential at these doses (He et al., 2025).

Collectively, although research on the structure–function relationship of BSPs has burgeoned, comprehensive toxicological profiling remains nascent. Despite expanded preclinical datasets from cellular and animal models, translational safety evidence for human applications remains critically underexplored. Moving forward, mechanistic *in vivo* toxicity studies must be prioritized to clarify hazard pathways and establish safety thresholds for BSP’s clinical translation.

## 8 Drug carrier applications

Drug carriers optimize targeted delivery efficiency by precisely controlling absorption, distribution, and release kinetics (Putro et al., 2023; Sun et al., 2020). As naturally derived biopolymers, polysaccharides (from plants, animals, and microorganisms) serve as ideal carrier materials due to their biocompatibility, biodegradability, and renewability. Structurally, polysaccharides contain abundant active groups amenable to chemical modifications for constructing vesicles, hydrogels, micelles, nanoparticles, and other advanced delivery systems that optimize physicochemical properties (Ramadan et al., 2025). Notably, polysaccharide exhibits exceptional potential as a next-generation carrier (Elkomy et al., 2022; Zhang et al., 2018), particularly for overcoming the low oral bioavailability of small-molecule drugs caused by poor solubility, gastrointestinal instability, and limited permeability (Peppas et al., 2009). BSP-based delivery systems enhance drug loading capacity, enable controlled release, and significantly improve bioavailability, with the fundamental goal of achieving spatiotemporally precise drug distribution at target sites. Current applications of BSP carriers are systematically summarized in Table 4.

**TABLE 4:** Studies of *B. striata* polysaccharides as drug carrier applications.

No	Polymer	Type of drug carrier	Cross-linker	Loaded drug	Target	Specificity	Reference
1	OBGTP	Hydrogel (Figure 10)	N/A	Gelatin/tea polyphenol	Liver and skin	Adhesive properties and good biocompatibility	Ma et al. (2024)
2	BSP/KGM	Hydrogel	Hydrogen bonding	N/A	L929 cell	Increasing the water-holding capacity, improving the swelling degree, and enhancing the mechanical properties	Shang et al. (2023)
3	BSP/CMC/CBM_940_	Hydrogel	Triethanolamine	N/A	Wound	Porous structure, high elastic property, and high-water retention	Huang et al. (2019)
4	BSP	Hydrogel	N/A	N/A	Wound	Good viscoelasticity, physical strength, and improved skin permeability	Cui et al. (2017)
5	BSP/CS/β-GP@SDSS	Hydrogel	N/A	Danshensu	Wound	Good water absorption and bonding properties	Gao et al. (2025)
6	CCHG/BSP	Hydrogel	Carbomer 940 and carboxymethyl chitosan	N/A	Wound	Good water retention ability	Li et al. (2024b)
7	WPU-BSP	Waterborne polyurethane hydrogel	N/A	N/A	Wound	Good compressive strength, water absorption, and retention ability	Chen et al. (2023b)
8	M8Bx	Hydrogel	Methylcellulose and methylparaben	N/A	Wound	Biocompatible with live tissue	Jakfar et al. (2022)
9	BSP-PAM semi-IPN PhCs	Photonic crystal hydrogel	N/A	N/A	N/A	Good stimulus responsiveness	Sun et al. (2022)
10	BSP/BER	Hydrogel (Figure 11)	Carbomer 940	Berberine	Wound	Straightforward preparation process, remarkable biocompatibility, hydrophilicity, and adhesion properties	Hu et al. (2023)
11	N/A	Hydrogels	1,4-butanediol diglycidyl ether	N/A	Wound	Biocompatible and biodegradable, good water retention, high swelling capacity, greater thermal stability, and superior mechanical properties	Qiu et al. (2024b)
12	OBSP-CS-LP	Hydrogels	Chitosan	Probiotic-bound	N/A	Synergistic antibacterial abilities and low toxicity	Yang et al. (2020)
13	BSP-g-PAA/PVA DN	Hydrogel	N/A	N/A	Hepatic	Enhancing the mechanical properties, rapid hemostasis, and non-cytotoxic	Xiang et al. (2022b)
14	N/A	Hydrogel	N/A	N/A	Skin	Good viscoelastic characteristics, rapid hemostasis, and shortened wound healing time	Zhang et al. (2023)
15	HTFC@BSP-20	Hydrogel	Cyclodextrin-ferrocene unit	N/A	Skin	Outstanding antibacterial properties and favorable biocompatibility	Zhang et al. (2024b)
16	TMP-BSP-HA	Hydrogel	Hyaluronic acid	Tetramethylpyrazine	Diabetic wound	High swelling and sustained drug release, accelerating wound healing	Zhang et al. (2024a)
17	PUB	Hydrogel	Polyurethane chains	N/A	Wound	High hemostatic ability, high absorption to exudates, softness, non-toxicity, and non-irritating property	Chen et al. (2022)
18	PBBT	Dual-dynamic bonds, crosslinked hydrogel	Tannic acid	N/A	Liver	Excellent mechanical strength, mechanical stability, rapid self-healing ability, and pH- and sugar responsiveness	Zhang et al. (2024c)
19	BSP-MA	Cryogel scaffold	2-morpholinoethanesulfonic acid, N-hydroxysuccinimide, and 1-ethyl-3-(3-dimethylaminopropyl) carbodiimide hydrochloride	N/A	RAW 264.7 cell	Good cytocompatibility	Chen et al. (2021b)
20	(HA)/BSP-182	Hydrogel	Hydrogen bonding	Hyaluronic acid	N/A	Good rheological properties, textural attributes, and thermal stability	Ma et al. (2025)
21	PB-ISA/BSP	Thermosensitive gel (Figure 12)	N/A	N/A	Bacteria	High photothermal conversion efficiency	Zeng et al. (2023)
22	CVO-NEGs	Gel	Nanoemulsion	Chamomile volatile oil	Skin	Promote immune response and mitigate inflammation	Xu et al. (2024)
23	AG@BSP-VES	Nanomicelle	N/A	Andrographolide	Colon and CT26 cells	Low hemolysis rate, exhibits excellent biological safety, strong drug carrying capacity, and high biocompatibility, and enhances the internal and external antitumor effect	Yue et al. (2024)
24	CS-AS-BSP MNs	Microneedles	N/A	Asiaticoside	N/A	Continuous drug release and good wound healing	Lv et al. (2023)
25	EGF@BSP-CeO2/PLGA	Nanofibrous scaffolds (Figure 13)	Electrospinning emulsion	N/A	Wound	Excellent antioxidant and antibacterial effects	Zhao et al. (2024a)
26	RA-BSP-PVA@PLA	Electrospinning nanofiber	Coaxial electrospinning	Rosmarinic acid	Wound	Suitable air permeability, excellent flexibility, good flexibility, and better accommodates wounds	Zhong et al. (2023)
27	OME-BSP	Nanoparticle	Inverse emulsion and surface cross-linking	Omeprazole	Gastric Ulcer	Spherical, uniformly dispersed, small in size, and with good drug loading	Li et al. (2023)
28	His-SA-BSP	Nanoparticles (Figure 14)	N/A	Doxorubicin	MCF-7 cells	Good biocompatibility and enhances antitumor effect	Zhu et al. (2018)
29	HA-SH-zein NPs	Hydrogel, nanoparticles	N/A	Puerarin	Colon	Enhancing the retention time of drugs in colon and effectively controlling drug release	Zhao et al. (2024c)
30	CT/AgB-MNs	Microneedle, silver nanoparticle	N/A	N/A	L929 cell	Promoted wound healing, enhance the internal and external antibacterial effects	Yang et al. (2022)
31	OVA-BMNs	Microneedle (Figure 15)	N/A	Antigen ovalbumin	Skin	Good cell compatibility, low bacterial skin permeability, slight irritation to the skin, and no infection or inflammation	Zhou et al. (2022)
32	BCP-MNs	Microneedle	N/A	Peony leaf extract	NIH-3T3 fibroblasts	Good mechanical properties, stability, and biocompatibility, potent antioxidant effects	Ye et al. (2024)
33	His-SA-BSP	Micelles	N/A	Doxorubicin	MCF-7 cells	Satisfactory encapsulation efficiency, loading capacity, and drug compatibility	Wang et al. (2019a)
34	BSP-ss-SA	Micelles	N/A	Docetaxel	HepG2 cells and 4T1 cells	Enhance anti-tumor drugs release at tumor sites and improve the therapeutic effect	Liu et al. (2020)
35	mCSB@TA	Microspheres	Tannic acid	N/A	Wound	Good swelling properties, sustainable release, good long-lasting antibacterial properties	Wang et al. (2022)
36	CS/Alg/Bsp	Microspheres	Chitosan	N/A	3T3 cells	Good spherical shape, abundant surface pores, and good dispersibility	Wang et al. (2017)

N/A, not available.

### 8.1 Hydrogel

Schiff-base crosslinked BSP hydrogels represent advanced therapeutic platforms that integrate structural advantages, including ECM-mimetic biocompatibility, stress-responsive self-repair, and renewable carbohydrate sourcing (over 90% natural carbohydrate abundance) (Xu J. et al., 2019). Similarly, these systems demonstrate exceptional promise in advanced wound management through pH/O_2_ microenvironment regulation, microbial barrier establishment, and spatiotemporal drug release (Huang et al., 2019; Yi et al., 2021). Innovative hydrogel systems demonstrate diabetic wound healing efficacy, Zhao et al. (2024b) constructed a self-healing hydrogel matrix by crosslinking oxidized *B. striata* polysaccharide (OBSP) and quaternized chitosan (HACC) with Schiff base and integrated photothermal response CuO@BER nanoparticles, developing multifunctional composite hydrogels (CuO@BER/BH). This system achieves efficient eradication of 99.2% of *Staphylococcus aureus* through near-infrared triggered synergistic release of berberine and targeted photothermal effect; synchronously regulates the wound microenvironment, significantly eliminating DPPH free radicals; downregulates the inflammatory factor TNF-α by 53.8%; and promotes angiogenesis, thus driving the healing rate of diabetic wounds to 85% within 10 days. Its outstanding self-healing, injectability, and dynamic wound adaptability provide a new intelligent solution for clinical prevention of bacterial invasion and accelerated tissue regeneration. Similarly, to resolve multifactorial dysregulation in chronic wound microenvironments. Ma et al. (2024) employed an innovative Schiff-base/hydrogen bond dual-network to crosslink OBSP with ADH-modified gelatin (Gel-ADH), constructing a multifunctional hydrogel (OBGTP) loaded with tea polyphenols (TPs). This platform synergistically promotes wound resolution via autonomous self-repair and controlled TP release, demonstrating kinetics demonstrating potent antioxidant activity (89.7% DPPH clearance) and broad-spectrum antimicrobial action (over 99% eradication) while significantly enhancing fibroblast migration (91.47% scratch wound recovery at 48 h) with 53.8% suppression of pro-inflammatory TNF-α, collectively driving 98.4% resolution of infected wounds in rat models by day 14. The hydrogel orchestrates sequential regenerative phases: rapid hemostasis, inflammation modulation, and matrix remodeling, establishing a pioneering injectable platform for comprehensive management of diabetic ulcers and recalcitrant wounds. In addition, Gao et al. (2025) engineered BSP composite hydrogels via thermoresponsive crosslinking in a CS/β-GP matrix, incorporating SDSS. *B. striata* polysaccharide water gel possesses a microporous structure (pore size 10–30 μm), high swelling rate (169.47% ± 4.54%), and slow-release features (cumulative SDSS release reached 96.26% ± 2.57% over 53 h). It accelerates wound healing by promoting fibroblast proliferation, enhancing collagen expression, and inhibiting *S. aureus* by > 90%. On day 7, the hydrogel group showed a 27.61% higher healing rate than the control (*p* < 0.05), and histological analysis on day 16 indicated improved epithelial regeneration and collagen deposition, showcasing its potential in chronic wound treatment.

Collectively, these hydrogel platforms achieve 85%–98.4% infected wound resolution through multimodal mechanisms: synergistic photothermal/antibiotic action, potent antioxidant/anti-inflammatory effects, and enhanced collagen deposition/angiogenesis, establishing a transformative strategy for comprehensive diabetic wound management.

### 8.2 Microneedles

BSP-based microneedles achieve triple therapeutic breakthroughs by employing material composition strategies (CMCH/chitosan reinforcement of mechanical strength), innovative drug-loading techniques (HP-β-CD encapsulation of hydrophobic drugs), and functional synergy design (combined antibacterial/anti-fibrotic action). It provides a transdermal delivery solution, balancing high efficacy, safety, and clinical adaptability, particularly suited for vaccine delivery, infected wound regeneration, and pathological scar therapy. For example, Zhou et al. (2022) engineered *B. striata* polysaccharide microneedles (BMNs) via polydimethylsiloxane (PDMS) micromolding and centrifugal casting. Vaccine delivery capabilities were characterized through *ex vivo* permeation studies in Sprague–Dawley rat skin using Franz diffusion cells, with preliminary analysis of the physical encapsulation-controlled release mechanism. The results revealed (1) exceptional fracture resistance (0.63 N/needle), outperforming HA/PVA microneedles; (2) rapid in-skin dissolution (<60 min); (3) enhanced microbial barrier function (20 × lower bacterial penetration vs. subcutaneous injection); and (4) preserved OVA structural stability over 21 days with 76.7% cumulative transdermal release within 3 h. Yang et al. (2022) engineered chitosan/*B. striata* polysaccharide composite microneedles (CT/AgB MNs) through *in situ* AgNP synthesis. These demonstrated 1) effective biofilm penetration (0.21 N fracture force); 2) synergistic antibacterial/antioxidant action (99.99% MRSA eradication at 0.9 μg/mg Ag; fourfold DPPH clearance enhancement); and 3) accelerated wound healing (37% fibroblast expansion at 72 h; 98.2% resolution by day 15). Mechanistically, these effects correlated with ROS clearance, TNF-α suppression, and VEGF/EGF induction. Zhang N. et al. (2022) engineered CMCH/*B. striata* polysaccharide composite microneedles incorporating HP-β-CD-complexed triamcinolone acetonide and verapamil, demonstrating (1) enhanced mechanical strength (1.28 N/needle, +49% vs. unloaded) with 200 μm dermal penetration; (2) synergistic hypertrophic scar (HS) reduction (48.7%, *p* < 0.05 vs. monotherapy); (3) mechanistic TGF-β1 suppression (61.2%) and hydroxyproline decrease (53.8%). This pioneering work established BSP/CMCH mechanical reinforcement synergy at optimized drug loading (0.059 mg TA/patch; 0.042 mg VRP/patch).

Collectively, these microneedle platforms enable precision transdermal delivery through mechanical reinforcement and dissolution kinetics optimization, resolve infected wounds via synergistic antibacterial/antioxidant action, and inhibit pathological scars by suppressing TGF-β1 signaling and hydroxyproline deposition, establishing a clinically adaptable platform for targeted dermatotherapy.

### 8.3 Nanoparticles

BSP-based nanoparticles leverage nanoscale dimensions and EPR effects for tumor targeting, while functional modifications enable versatile therapeutic loading. Three advanced platforms demonstrate targeted applications: tumor-targeted nanomicelles, core-shell nanofibers, and anti-dermatitis nanoemulsions. BSP-VES amphiphilic polymer was synthesized via esterification, and andrographolide-loaded nanomicelles (AG@BSP-VES, 84.68 ± 2.08 nm diameter, 6.84% drug loading) were prepared by dialysis. This system exhibited low hemolysis (<5%), sustained release (47.6% cumulative release at 24 h), and tumor targeting (EPR effect) (Yue et al., 2024). *In vitro* studies demonstrated its specific enhancement of AG internalization in colon cancer cells (CT26) (3-fold uptake increase) and a significant reduction in AG’s IC50 value (11.69 vs. 31.16 μg/mL). *In vivo* imaging revealed high accumulation in subcutaneous/orthotopic colon tumors (55.37%/17.31% ID/g), confirming its efficacy through elevated drug concentration at target sites and highlighting clinical potential for natural polysaccharide-based targeted drug delivery against colon cancer. Core-shell nanofibers (RA-BSP-PVA@PLA; diameter 0.69 ± 0.09 μm) were fabricated via coaxial electrospinning using polylactic acid (PLA) as the shell and polyvinyl alcohol (PVA) loaded with *B. striata* polysaccharide and rosmarinic acid (RA) as the core (Zhong et al., 2023). The material exhibited high hydrophobicity (contact angle 129.10° ± 2.08°), optimal water vapor transmission rate (555.29 ± 7.02 g/m^2^/day), and mechanical strength (tensile strength: 6.31 ± 0.10 MPa; elongation at break: 76.62% ± 4.51%). Studies demonstrated its acceleration of wound healing by promoting M1-to-M2 macrophage polarization (CD206^+^ cells increased to 48.6%) and downregulating MPO^+^ inflammatory factors (64.2% reduction at day 5), achieving 97.43% wound closure in rat models within 15 days, highlighting clinical potential as advanced wound dressing. Xu et al. (2024) prepared chamomile volatile oil nanoemulsions (CVO-NEs; 19.07 ± 0.28 nm) via the phase transition method and encapsulated them with BSPs to form gels (CVO-NEGs). This system exhibited shear-thinning properties (92.8% viscosity reduction) and a porous network structure, which regulated CD4^+^ T cell differentiation (46.8%–52.3% reduction in Th2/Th17 cells) and reduced inflammatory factors (57.2%–63.4% decrease in serum TNF-α/IL-4), significantly improving atopic dermatitis lesions in mice (a 48.3% reduction in epidermal thickness and a 64.2% decrease in mast cell infiltration).

Overall, these platforms exemplify BSP’s versatility in nanocarrier design, from tumor-targeted chemotherapy and advanced wound dressings to immunomodulatory dermatitis therapy, establishing a transformative bridge between natural polysaccharides and precision medicine.

### 8.4 Microspheres

Microsphere drug carriers have been developed for four decades, offering core advantages over traditional systems, including enhanced drug stability and controlled release. Building upon this foundation, Wang et al. (2022) engineered innovative calcium alginate/silk fibroin peptide/BSP composite microspheres (mCSB) using reverse emulsion fabrication. Their high swelling capacity (1,188.09%) accelerates thrombus formation by significantly increasing platelet aggregation index (1.5) and erythrocyte aggregation rate (80%). When loaded with tannic acid (TA), hydrogen-bonding interactions enable sustained release (86.96% over 96 h), conferring long-lasting antibacterial and anti-inflammatory activities, thereby establishing a novel approach for multifunctional wound dressing design.

### 8.5 Copolymer micelles

Amphiphilic polymers self-assemble in aqueous solutions above critical micelle concentration (CMC), forming core-shell nanostructures with hydrophobic cores for drug encapsulation and hydrophilic coronae for colloidal stability (Gong et al., 2010; Kalhapure and Renukuntla, 2018). BSP-based micelles demonstrate enhanced therapeutic efficacy through stimulus-responsive designs. Wang C. et al. (2019) developed histidine-stearic acid functionalized BSP micelles (His-SA-BSP), exhibiting dual-stage pH sensitivity: charge reversal at pH 6.5 enhanced cellular uptake by 42% (*p* < 0.05), while micelle disintegration at pH 5.0 triggered rapid doxorubicin release (70% within 36 h), achieving 62% tumor suppression in MCF-7 xenografts. Liu et al. (2020) developed redox/pH-dual responsive micelles (BSP-ss-SA), achieving 87.25% docetaxel release at pH 5.0 within 3 h and 70.53% tumor suppression in 4T1-bearing mice. Wang et al. (2021) demonstrated folate-modified micelles (FA-BSP-SA) bind bovine serum albumin via static quenching (KSV = 8.15 × 104 L/mol), forming a protein-corona that reduces cellular uptake by 42% and attenuates antitumor efficacy. Although stimulus-responsive BSP micelles significantly enhance antitumor efficacy through precision drug delivery, protein corona formation remains a critical barrier to clinical translation.

## 9 Potential applications for *B. striata* polysaccharides

The tuber of *B. striata* has been employed in traditional medicine across China and East Asia for hundreds of years, particularly for wound healing, reducing swelling, and promoting tissue regeneration (Chen Z. Y. et al., 2020). In the 21st century, advances in pharmacological research have identified BSP, a glucomannan featuring a backbone of (1→4)-β-D-mannopyranosyl and (1→2)-α-D-glucopyranosyl linkages as the key constituent responsible for its wide range of biological activities (Zhang et al., 2019). BSP has been extensively validated for its outstanding biocompatibility, biodegradability, low toxicological risk, and notable functionalities, including anti-inflammation, coagulation-promoting, antioxidation, and tissue-regenerative effects (Jiang et al., 2013; Xu D. et al., 2019; Yang et al., 2019). These advantageous properties have garnered significant attention for BSP as a versatile functional biomaterial with considerable potential in biomedical and material science applications.

In recent years, research has increasingly centered on formulating BSPs with other natural or synthetic macromolecules to optimize the mechanical strength, hydration capacity, and biofunctionality of hydrogels. For example, hydrogels prepared by blending BSPs with konjac glucomannan exhibit favorable biocompatibility and enhance wound-healing processes (Shang et al., 2023). It is well-established that persistent or excessive inflammation at the early stage of wound healing often leads to repair failure. BSPs potently attenuate inflammation, specifically via the suppression of NLRP3 inflammasome activation (Zhao et al., 2021). Moreover, BSPs promote angiogenesis by stimulating vascular endothelial cell proliferation and migration and upregulating VEGF expression, thereby ensuring sufficient oxygenation and nutrient delivery to support tissue regeneration (Chen Z. Y. et al., 2020; Liu J. et al., 2022).

Owing to its favorable biocompatibility and biodegradable nature, BSP is also regarded as a promising candidate for drug delivery systems. The release kinetics of encapsulated drugs serve as a crucial metric for carrier performance. *In vitro* release studies, often modeled using approaches such as the Korsmeyer–Peppas equation, elucidate underlying release mechanisms (diffusion-, erosion-, or swelling-controlled) from BSP matrices (Bayer, 2023; Illanes-Bordomás et al., 2023; Liang et al., 2021). Fine-tuning parameters such as crosslinking density, composite blending ratio, or incorporation of stimuli-responsive elements allows precise control over release rates and modes, enabling customizable profiles, including zero-order and pulsatile release, tailored to specific clinical applications.

Apart from wound repair and programmable drug delivery, BSP’s multifaceted bioactivities indicate broader potential. In oral healthcare, BSP-based mucoadhesive films have been developed for recurrent aphthous ulcers, capitalizing on their anti-inflammatory, analgesic, and pro-regenerative properties to enable prolonged localized delivery and shorten healing duration (Liao et al., 2019). In dermatology and cosmetics, the antioxidant, anti-inflammatory, and collagen-synthesis-promoting effects of the BSP make it a valuable ingredient in anti-aging and functional skincare products (Wang et al., 2006). It can be incorporated into serums, creams, or masks as a natural and safe active ingredient to repair damaged skin, reduce fine lines, and improve skin elasticity (He X. et al., 2024; Song et al., 2017). In the food industry, the BSP serves as a natural and safe polysaccharide with potential as a functional food additive or edible coating (Zhang et al., 2019; Zhu et al., 2023). Although research in this area remains limited, its antioxidant properties may find applications in food preservation or health-promoting products (Zhai et al., 2021).

In summary, *B. striata* polysaccharide, a natural polymer originating from traditional Chinese medicine, exhibits considerable promise in the biomedical field, as evidenced by broad and thorough experimental confirmation. Nevertheless, there remains a clear recognition that the translational research of the BSP continues to encounter significant obstacles. The most prominent issue is the translational gap from laboratory research to clinical application. Table 5 summarizes experimental products containing BSPs, along with their asserted biological properties. Although substantial preclinical studies (including *in vitro* cell experiments and animal models) have confirmed its efficacy and safety, to date, no registered clinical trials or commercially available products have been identified for the aforementioned advanced applications, such as BSP-based hydrogel dressings or drug delivery systems.

**TABLE 5:** Patents related to *B. striata* polysaccharide products.

NO	Application	Main composition	Pharmacological property	Publish number
1	Skincare products	Collagen, dihydromyricetin, *Bletilla striata* polysaccharides	Beauty and whitening	CN105919841A
2	Tooth protection	*Bletilla striata* polysaccharides, emodin, menthol	Gum inflammation, gum bleeding, oral ulcers	CN109276475A
3	Pharmaceutical	*Centella asiatica*, *Bletilla striata* polysaccharides	Scar inhibition and promotion of wound healing	CN115414381A
4	Pharmaceutical	Alpinia officinarum Hance, Eupatorium fortune Turcz, Artemisia capillaris Thunb., Phyllanthus urinaria L., *Bletilla striata* polysaccharides	Chicken glandular gastritis	CN114699496A
5	Pharmaceutical	Tiliroside, *Bletilla striata* polysaccharides, fluocinolone acetonide	Dermatitis	CN112252297A
6	Functional materials	*Bletilla striata* polysaccharides, sea buckthorn oil, Forsythia oil, Angelica seed oil, asiaticoside	Cervical erosion	CN106692307A
7	Functional materials	Tannic acid, AgNO_3_, *Bletilla striata* polysaccharides	Promote wound healing	CN114668778A

## 10 Conclusion and prospects

BSP, a primary bioactive compound from *Bletilla striata*, exhibits multifaceted biological activities, including immunomodulation, antioxidant, anti-inflammatory, and antitumor effects. Advances in extraction, purification, structural characterization, and mechanistic studies have propelled BSPs into the spotlight of pharmaceutical research. Although current studies have elucidated primary structures (monosaccharide composition, molecular weight, and glycosidic linkages), advanced structural features (e.g., spatial conformation, structure-activity relationships) remain underexplored due to structural complexity and technical limitations. Moreover, BSP serves as a versatile platform for multifunctional drug delivery systems, encompassing copolymer micelles, microspheres, nanoparticles, microneedles, and hydrogel matrices. Through strategic extraction optimization and formulation design, BSPs enable tailored therapeutic functionalities across diverse biomedical applications, facilitating novel therapeutic innovation pipelines and market diversification.

Hot-water extraction (HWE remains the predominant method for isolating BSPs due to its operational simplicity, economy, and safety). However, this method exhibits critical limitations: low extraction efficiency, prolonged duration, and degradation of thermolabile constituents. To overcome these drawbacks, emerging techniques, including ultrasonic assisted extraction (UAE and MAE), are increasingly employed. Although these improve yields and minimize solvent usage, they also carry risks of polysaccharide depolymerization. Thus, development of sustainable, high-efficiency extraction technologies is an urgent priority.

A key parallel challenge lies in purification: conventional deproteinization approaches (Sevage/enzymatic protocols) incur over 30% polysaccharide loss, hindering acquisition of high-purity BSPs (>95%). Moreover, BSP monosaccharide profiles display significant method-dependent variation. Although primary structural features, such as monosaccharide composition, molecular weight, and glycosidic linkages, are characterized, higher-order structural analysis is impeded by conformational heterogeneity from irregular branching and molecular flexibility (<10% resolved). This limitation is compounded by broad molecular weight dispersity (0.94–722.90 kDa) and monosaccharide heterogeneity (Glu, Man, etc.), thereby challenging batch-to-batch reproducibility. Notably, chemical modifications can structurally remodel BSPs, directly modulating their bioactivity and drug delivery performance. Pharmacologically, BSPs demonstrate confirmed bioactivities, including potent antioxidant effects (>80% ROS scavenging) and immunomodulation (2.5-fold macrophage activation enhancement). Nevertheless, systematic investigation is still lacking to elucidate their mechanistic regulation of key pathways (e.g., NF-*κ*B/TLR4). Crucially, persistent knowledge gaps include quantitative structure–activity relationships and mechanistic actions. For instance, although inverse correlations exist between molecular weight and antioxidant potency, predictive quantitative models remain undeveloped.

Translational barriers further hinder applications: native BSPs face enzymatic degradation susceptibility and low oral bioavailability (<5%). Even nanocarriers (micelles/microneedles) suffer 42% reduced cellular uptake due to protein corona formation. Despite therapeutic promise in gastrointestinal disorders achieved through microbiota modulation, metabolite regulation, and intestinal barrier protection (positioning BSPs as natural prebiotic-based gastroprotectants), clinical translation is impeded by the absence of long-term toxicity profiles and human ADME data. To resolve these challenges, future work should implement the following.

1. Develop green extraction technologies (ionic liquid-assisted/supercritical fluid extraction) targeting 40% yield enhancement with a 60% solvent reduction.

Implement scalable purification via membrane separation (nanofiltration/ceramic) and preparative HPLC to achieve >98% purity. Integrate advanced structural tools, containing high-field NMR, cryo-EM, and AI-driven molecular dynamics, to elucidate tertiary structures and folding dynamics.

2. Employ multi-omics integration (proteomics for target deconvolution, metabolomics for pathway mapping) to delineate regulatory networks (e.g., the microbiota–SCFA–immune axis).
3. Establish predictive QSAR models correlating molecular weight/branching with bioactivity to enable rational drug design.
4. Expand sustainable sourcing by characterizing leaf/flower-derived polysaccharides for conserved bioactivity, supporting circular bioeconomy models.
5. Conduct comprehensive preclinical studies—acute/chronic toxicity (LD_50_, organ accumulation) and PK (C_max_/T_max_) progressing toward Phase I trials.
6. Engineer advanced delivery systems via PEGylation/biomimetic surfaces (reducing protein corona by 50% decreased association).

Collectively, these integrated strategies will overcome existing barriers and unlock BSP’s full therapeutic potential.
