# Supplementary material for: Recent advances in Bletilla striata polysaccharide research: extraction methodologies, structural elucidation, pharmacological mechanisms, structure–activity relationships, and therapeutic delivery applications
Source: Front Pharmacol. 2025 Nov 5;16:1688676. doi: 10.3389/fphar.2025.1688676 (PMC12627022; doi:10.3389/fphar.2025.1688676)
Supplement: Supplementary file 1 [file Table1.docx]

**Supplementary Data**

Table 1 Extraction and purification of *Bletilla striata* polysaccharide.

| Name | Extraction | Pretreatment | Time(min) | Temp (℃) | Power | Solid-liquid ratio | Optimization method | Chemical composition analysis | total sugar content (%) | Purification methods | Ref |
| --- | --- | --- | --- | --- | --- | --- | --- | --- | --- | --- | --- |
| BSP | HWE | The tubers were dried in a vacuum drying oven at 45 °C, then crushed sieved with a 30-mesh  sieve prior to extraction with petroleum ether and  ethanol | 120 | 70 | N/A | 1:40 (m/v) | N/A | N/A | N/A | Sevage protein removal, Ethanol precipitation, | (Li et al., 2021) |
| BSP | HWE | The tubers were dried in an oven, then extracted with 95 % ethanol and petroleum  ether | 240 | 75 | N/A | 1:40 (m/v) | N/A | N/A | N/A | Sevage protein removal, Ethanol precipitation, | (Ma et al., 2024) |
| BSP |  | The tubers were dried in an oven, then crushed sieved with a 30-mesh  Sieve; The powder was degreased in petroleum ether and refluxed ethanol | 120 | 70 | N/A | 1:40 (m/v) | N/A | N/A | N/A | Sevage protein removal, Ethanol precipitation | (Chen et al., 2019) |
| BSP | HWE | dried B. striata were ground | 240 | 80 | N/A | N/A | N/A | N/A | N/A | Sevage protein removal, Ethanol precipitation | (Chen et al., 2021b) |
| BSPs-H | HWE | pretreatment of Bletilla striata tuber powders using an ethanol solution | 180 | 70 | N/A | 1:30 | N/A | phenol sulfuric acid, Bradford method and m-hydroxybiphenyl method | 93.25 | Ethanol precipitation | (Chen et al., 2021a) |
| BSPs-B | HWE | pretreatment of Bletilla striata tuber powders using an ethanol solution | 180 | 100 | N/A | 1:30 | N/A | phenol sulfuric acid method, Bradford method and m-hydroxybiphenyl method | 85.41 | Ethanol precipitation | (Chen et al., 2021a) |
| BSP-1 | HWE | Powders were prepared, Powders were pre- extracted with 85% ethanol | 180 | 90 | N/A | N/A | N/A | phenol-sulfuric acid method | 71.4 | Ethanol precipitation, Sevage protein removal, DEAE-Cellulose-52 column, a Sephadex G-200 column | (Wang et al., 2019) |
| BSP-2 | HWE | Powders were prepared | 180 | 90 | N/A | N/A | N/A | phenol-sulfuric acid method | 81.2 | Ethanol precipitation, Sevage protein removal, DEAE-Cellulose-52 column, a Sephadex G-200 column | (Wang et al., 2019) |
| BSP | HWE | Powders were pre- extracted with 95% ethanol and petroleum ether | 120 | 70 | N/A | 1:40 | N/A | phenol sulfuric acid method, and Bradford method | 63.46 | Ethanol precipitation, Sevage protein removal, | (Liao et al., 2019) |
| BSP | HWE | - | 240 | 100 | N/A | N/A | N/A | phenol sulfuric acid method | 65.3 | Ethanol precipitation, Sevage protein removal, | (Zhang et al., 2019) |
| BP | HWE | Powders were prepared | 120 | 90 | N/A | 1:40 | N/A | phenol sulfuric acid, Bradford method and m-hydroxybiphenyl method | 78.69 | Ethanol precipitation, Sevage protein removal, | (Wang et al., 2023) |
| BSP | HWE | Powders were prepared; Powders were pre- extracted with 95% ethanol and petroleum ether | N/A | 70 | N/A | 1:40 | N/A | phenol sulfuric acid method | 90.18 | Ethanol precipitation, Sevage protein removal, | (Zhong et al., 2023) |
| BSP | HWE | Powders were prepared, | 240 | 100 | N/A | N/A | N/A | N/A | 98 | Ethanol precipitation | (Zhang et al., 2025) |
| BSP | HWE |  | 240 | 80 | N/A | N/A | N/A | N/A | N/A | Sevage protein removal, Ethanol precipitation | (Thacker et al., 2020) |
| BSP | HWE | Powders were prepared, | 210 | 80 | N/A | 1:40 | N/A | phenol sulfuric acid method | 94.2 | Ethanol precipitation, carbon decolorized, enzymatic hydrolysis and  Sevag reaction protein removal, DEAE-cellulose, Sephadex G-200 | (Cui et al., 2017) |
| BSP | HWE | Powders were prepared, | 480 | N/A | N/A | 1:110 | N/A | phenol-sulfuric acid method, BCA protein assays | 99.21 | Ethanol precipitation | (Zhao et al., 2021) |
| BSP | HWE | Powders were prepared | 240 | 80 | N/A | 1:100 | N/A | phenol-sulfuric acid method | 96.4 | Ethanol precipitation, Sevage protein removal, dialyzed, DEAE-52 cellulose  column, | (Wang et al., 2024b) |
| BSP | HWE | N/A | 240 | 80 | N/A | 1:10 | N/A | phenol-sulfuric acid, bicinchoninic acid and m-hydroxydiphenyl methods | 93.28 | Ethanol precipitation, freezing and thawing protein removal, DEAE-52 cellulose column, Sephadex G-150 column | (Wang et al., 2020) |
| BSPS | HWE | N/A | 240 | 100 | N/A | N/A | N/A | Phenol sulfuric acid, carbazole sulfuric acid, BaCl2 gelatin turbidity, and hydroxylamine colorimetry method | 98.09 | Ethanol precipitation, Sevage protein removal, Sephadex  G200 column, DEAE-Sephadex A-50 column | (He et al., 2024) |
| BSP-182 | HWE | Powders were prepared | N/A | 90 | N/A | 1:10 | N/A | N/A | N/A | Ethanol precipitation, Sevage protein removal, DEAE-52  Cellulose, automated gel purification system | (Ma et al., 2025) |
| pFSP | HWE | fresh Bletilla striata were dried and then crushed into powders, petroleum  ether fats removal, dialysis | 120 | 80 | N/A | 1:50 | N/A | Phenol sulfuric acid, Bradford method | 97.82 | Ethanol precipitation, 15% trichloroacetic acid protein removal, DEAE-Sepharose fast flow gel column | (Chen et al., 2020) |
| BSPb | HWE | N/A | 180 | 60 | N/A | N/A | N/A | Phenol sulfuric acid | 99.36 | Ethanol precipitation, Sevage protein removal, DEAE (Cl−) -cellulose column, DEAE (OH−) -cellulose column | (Wang et al., 2014) |
| LMW-BSP | HWE | N/A | 1440 | 4 | N/A | 1:15 | N/A | Phenol sulfuric acid, coomassie brilliant blue method, carbazole sulfuric method | 90.99 | Ethanol precipitation, Sevage protein removal | (Liu et al., 2022) |
| BSP | HWE | Powders were prepared | 180 | 70 | N/A | 1:20 | N/A | N/A | N/A | Ethanol precipitation, Sevage protein removal, DEAE and G-200 columns | (Zhu et al., 2018) |
| BSP | HWE | Powders were prepared and then pre- extracted with petroleum ether | 90 | 80 | N/A | 1:30 | N/A | N/A | N/A | Ethanol precipitation, activated carbon decolorization, dialyzed, DEAE-52  cellulose resin chromatography column, G-150  dextran gel column | (Zhao et al., 2025) |
| BSP | HWE | Powders were prepared and then pre- extracted with ethanol and petroleum ether | 240 | 70 | N/A | N/A | N/A | N/A | 84.42 | Ethanol precipitation, Sevage protein removal, DEAE-52 cellulose column | (Qiu et al., 2024b) |
| BSP | HWE | Powders were soaked in ethanol | 240 | 70 | N/A | N/A | N/A | Phenol sulfuric acid, coomassie brilliant blue method, AOAC method 996.11 | 89.3 | Ethanol precipitation, dialyzed, Repeated freeze-thaw cycles with α-amylase | (Hou et al., 2023) |
| BSP | HWE | N/A | 240 | 80 | N/A | N/A | N/A | Phenol sulfuric acid | 68 | Ethanol precipitation, dialyzed, Sevage protein removal | (Lai et al., 2018) |
| BSP1-1 | HWE | N/A | N/A | 60 | N/A | 1:40 | N/A | Phenol sulfuric acid, 3, 5-dinitrosalicylic acid colorimetry method, Bradford method | 85.56 | Ethanol precipitation, Sevage protein removal, dialyzed, DEAE FF 16/10 anion-exchange column, Sepharose CL-6B gel chromatography, | (Li et al., 2025) |
| pBSP | HWE | Powders were soaked in petroleum ether | 120 | 80 | N/A | N/A | N/A | Phenol sulfuric acid, | 98.7 | Ethanol precipitation, trichloroacetic acid protein removal, DEAE Sepharose Fast Flow gel column | (Xu et al., 2021) |
| BSP | HWE | Powders were pre- extracted with ethanol and petroleum ether | 240 | 80 | N/A | 1:30 | N/A | N/A | N/A | Sevage protein removal, DEAE-52 cellulose, Sephadex G-100 columns | (Jiang et al., 2023) |
| BVPS | HWE | Powders were prepared | 210 | 80 | N/A | 1:50 | N/A | Phenol sulfuric acid, coomassie brilliant blue method, 3, 5-dinitrosalicylic acid colorimetry method | 64.09 | Ethanol precipitation | (Kong et al., 2015) |
| BFPS | HWE | Powders were prepared | 210 | 80 | N/A | 1:50 | N/A | Phenol sulfuric acid, coomassie brilliant blue method, 3, 5-dinitrosalicylic acid colorimetry method | 65.64 | Ethanol precipitation | (Kong et al., 2015) |
| BSP-H | HWE | Powders were soaked in petroleum ether and ethanol | 60 | N/A | N/A | 1:30 | N/A | N/A | N/A | Ethanol precipitation | (Han et al., 2025) |
| BSP | AAE | N/A | 30 | 50 | N/A | N/A | N/A | N/A | N/A | DEAE-52 cellulose  column | (Chen et al., 2024a) |
| BSPs-A | AAE | pretreatment of Bletilla striata tuber powders using an ethanol solution | 180 | 60 | N/A | 1:30 | N/A | Phenol sulfuric acid, 3, 5-dinitrosalicylic acid colorimetry method, Bradford method, m-hydroxybiphenyl method | 90.37 | Ethanol precipitation | (Chen et al., 2021a) |
| BSP | AAE | Fresh tubers  were washed and cut into slices | 60 | 70 | N/A | 1:30 | N/A | N/A | N/A | Ethanol precipitation, Sevage protein removal | (Zhai et al., 2021) |
| BSP-1 | AAE | Fresh tubers  were washed and cut into slices | 60 | 70 | N/A | 1:30 | N/A | Phenol sulfuric acid, m-hydroxybiphenyl method | 93.3 | Ethanol precipitation, Sevage protein removal, distilled, DEAE-52 cellulose column | (Zhai et al., 2021) |
| BSP-2 | AAE | Fresh tubers  were washed and cut into slices | 60 | 70 | N/A | 1:30 | N/A | Phenol sulfuric acid, m-hydroxybiphenyl method | 93.5 | Ethanol precipitation, Sevage protein removal, distilled, DEAE-52 cellulose column | (Zhai et al., 2021) |
| BSP-3 | AAE | Fresh tubers  were washed and cut into slices | 60 | 70 | N/A | 1:30 | N/A | Phenol sulfuric acid, m-hydroxybiphenyl method | 96.23 | Ethanol precipitation, Sevage protein removal, distilled, DEAE-52 cellulose column | (Zhai et al., 2021) |
| BSP-4 | AAE | Fresh tubers  were washed and cut into slices | 60 | 70 | N/A | 1:30 | N/A | Phenol sulfuric acid, m-hydroxybiphenyl method | 90.21 | Ethanol precipitation, Sevage protein removal, distilled, DEAE-52 cellulose column | (Zhai et al., 2021) |
| BSP-5 | AAE | Fresh tubers  were washed and cut into slices | 60 | 70 | N/A | 1:30 | N/A | N/A | N/A | Ethanol precipitation, Sevage protein removal, distilled, DEAE-52 cellulose column | (Zhai et al., 2021) |
| BSPs-A | AAE | Powders were pre- extracted with ethanol and petroleum ether | 167 | 52 | N/A | 1:30 | response surface methodology, Single-factor experiments, RSM-GA-ANN modeling analysis | phenol sulfuric acid method, 3,5-dinitrosalicylic acid colorimetry method, Bradford method, and m-hydroxybiphenyl method | 97.94 | Ethanol precipitation, | (Chen et al., 2023) |
| BSP | AAE | Powders were prepared and then pre- extracted with ethanol and petroleum ether | N/A | N/A | N/A | 1:30 |  | N/A | N/A | Ethanol precipitation, Sevage protein removal, DEAE-52 cellulose column, Sephadex G-200 column | (Chen et al., 2024b) |
| BO | Acid-assisted  extraction | N/A | 90 | 90 | N/A | 1:9 | N/A | phenol sulfuric acid method, 3, 5-dinitrosalicylic acid colorimetry method, Bradford method | 89.27 | - | (Wang et al., 2023) |
| BSP-M | MAE | Powders were soaked in petroleum ether and ethanol | 9 | N/A | 600 | 1:30 | N/A | N/A | N/A | Ethanol precipitation | (Han et al., 2025) |
| BSPs-U | UAE | pretreatment of Bletilla striata tuber powders using an ethanol solution | 30 | 50 | 600W | 1:30 | N/A | phenol sulfuric acid method, 3, 5-dinitrosalicylic acid colorimetry method, Bradford method, m-hydroxybiphenyl method | 83.37 | Ethanol precipitation | (Chen et al., 2021a) |
| pBSP1 | UAE | Powders were prepared, | 34 | N/A | 450 | 1:15 | N/A | anthrone sulfuric acid method, m-hydroxydiphenyl method, BCA kit | 80.29 | Ethanol precipitation, Sevage protein removal, DEAE-52 cellulose column, Sephadex G-100 column. | (Qiu et al., 2024a) |
| BSP | UAE | N/A | 10 | 80 | N/A | 1:20 | N/A | phenol sulfuric acid method | 96 | Ethanol precipitation, Sevage protein removal, Sepharose CL-6B column | (Zhang et al., 2014) |
| BSP-V | UAE | Powders were soaked in petroleum ether and ethanol | 60 | 30 | 250 | 1:30 | N/A | N/A | N/A | Ethanol precipitation | (Han et al., 2025) |
| BSP | UCE | Bletilla striata were dried and then crushed into powders, | 30 | 70 | N/A | 1:30 | N/A | external standard method, | 94.6 | Ethanol precipitation, Sevage protein removal, AB-8 macroporous adsorption resin, dialyzed, gel purification column | (Huang et al., 2024) |
| FBP | MFE | Bacillus licheniformis was inoculated into the fermentation buffer | 720 | 37 | N/A | N/A | N/A | phenol sulfuric acid method, 3,5-dinitrosalicylic acid colorimetry method, BCA method | 90.82 | Ethanol precipitation | (Wang et al., 2024a) |
| BSP | DESE | N/A | 47 | 78 | N/A | 1:25 | single-factor and Box–Behnken response surface | N/A | N/A | Ethanol precipitation | (Luo et al., 2023) |
| BSP | IAE | Powders were prepared and then pre- extracted with ethanol and petroleum ether | 150 | 70 | N/A | 1:53 | Single-factor design, Box-Behnken design (BBD) and response surface methodology | phenol sulfuric acid method | 88.96 | Ethanol precipitation, Sevage protein removal, DEAE-52 cellulose anion-exchange column, dialyzed | (Qu et al., 2016) |
| BSP-E | EAE | Powders were soaked in petroleum ether and ethanol | 90 | 65 | N/A | 1:30 | N/A | N/A | N/A | Ethanol precipitation | (Han et al., 2025) |

Abbreviations: Ultrasonic compound enzyme (UCE); Alkali-assisted extraction (AAE), Infrared-assisted extraction (IAE); Deep eutectic solvents extraction (DESE), Microbial fermentation extraction (MFE); N/A, not available.

Reference

Chen, H., Lin, C., Wu, Y., Wang, B., Kui, M., Xu, J., Ma, H., Li, J., Zeng, J., Gao, W., and Chen, K. (2024a). Protective effects of degraded Bletilla striata polysaccharides against UVB-induced oxidative stress in skin. *Int J Biol Macromol* **277**, 134462. <https://doi.org/10.1016/j.ijbiomac.2024.134462>

Chen, H., Wang, B., Li, J., Xu, J., Zeng, J., Gao, W., and Chen, K. (2023). Comparative study on the extraction efficiency, characterization, and bioactivities of Bletilla striata polysaccharides using response surface methodology (RSM) and genetic algorithm-artificial neural network (GA-ANN). *Int J Biol Macromol* **226**, 982-995. <https://doi.org/10.1016/j.ijbiomac.2022.12.017>

Chen, H., Wu, Y., Wang, B., Kui, M., Xu, J., Ma, H., Li, J., Zeng, J., Gao, W., and Chen, K. (2024b). Skin healthcare protection with antioxidant and anti-melanogenesis activity of polysaccharide purification from Bletilla striata. *Int J Biol Macromol* **262**, 130016. <https://doi.org/10.1016/j.ijbiomac.2024.130016>

Chen, H., Zeng, J., Wang, B., Cheng, Z., Xu, J., Gao, W., and Chen, K. (2021a). Structural characterization and antioxidant activities of Bletilla striata polysaccharide extracted by different methods. *Carbohydr Polym* **266**, 118149. <https://doi.org/10.1016/j.carbpol.2021.118149>

Chen, H. Y., Lin, T. C., Chiang, C. Y., Wey, S. L., Lin, F. H., Yang, K. C., Chang, C. H., and Hu, M. H. (2021b). Antifibrotic Effect of Bletilla striata Polysaccharide-Resveratrol-Impregnated Dual-Layer Carboxymethyl Cellulose-Based Sponge for The Prevention of Epidural Fibrosis after Laminectomy. *Polymers (Basel)* **13**. <https://doi.org/10.3390/polym13132129>

Chen, J., Lv, L., Li, Y., Ren, X., Luo, H., Gao, Y., Yan, H., Li, Y., Qu, Y., Yang, L., Li, X., and Zeng, R. (2019). Preparation and evaluation of Bletilla striata polysaccharide/graphene oxide composite hemostatic sponge. *Int J Biol Macromol* **130**, 827-835. <https://doi.org/10.1016/j.ijbiomac.2019.02.137>

Chen, Z., Zhao, Y., Zhang, M., Yang, X., Yue, P., Tang, D., and Wei, X. (2020). Structural characterization and antioxidant activity of a new polysaccharide from Bletilla striata fibrous roots. *Carbohydr Polym* **227**, 115362. <https://doi.org/10.1016/j.carbpol.2019.115362>

Cui, X., Zhang, X., Yang, Y., Wang, C., Zhang, C., and Peng, G. (2017). Preparation and evaluation of novel hydrogel based on polysaccharide isolated from Bletilla striata. *Pharm Dev Technol* **22**, 1001-1011. <https://doi.org/10.1080/10837450.2016.1221422>

Han, X., Liu, H., Zhang, Y., Zhang, Y., Song, Z., Yang, L., Liu, X., Yang, L., Wu, M., and Tan, L. (2025). The effects of different extraction methods on the structure and antioxidant properties of Bletilla striata polysaccharide. *Preparative Biochemistry & Biotechnology* **55**, 392-402. <https://doi.org/10.1080/10826068.2024.2419862>

He, Z., He, Y., Meng, X., Ge, Z., and Sun, H. (2024). Structural characteristics and wound-healing effects of Bletilla striata fresh tuber polysaccharide. *Int J Biol Macromol* **278**, 134679. <https://doi.org/10.1016/j.ijbiomac.2024.134679>

Hou, Y., Zhao, J., Yin, J., and Nie, S. (2023). Structural properties of Bletilla striata polysaccharide and the synergistic gelation of polysaccharide and xanthan gum. *Food Hydrocolloids* **142**. <https://doi.org/10.1016/j.foodhyd.2023.108843>

Huang, F., Fan, Y., Liu, X., Chen, Y., Huang, Y., Meng, Y., and Liang, Y. (2024). Structural characterization and innate immunomodulatory effect of glucomannan from Bletilla striata. *Int J Biol Macromol* **273**, 133206. <https://doi.org/10.1016/j.ijbiomac.2024.133206>

Jiang, G., Wang, B., Wang, Y., Kong, H., Wang, Y., Gao, P., Guo, M., Li, W., Zhang, J., Wang, Z., and Niu, J. (2023). Structural characteristics of a novel Bletilla striata polysaccharide and its activities for the alleviation of liver fibrosis. *Carbohydr Polym* **313**, 120781. <https://doi.org/10.1016/j.carbpol.2023.120781>

Kong, L., Yu, L., Feng, T., Yin, X., Liu, T., and Dong, L. (2015). Physicochemical characterization of the polysaccharide from Bletilla striata: effect of drying method. *Carbohydr Polym* **125**, 1-8. <https://doi.org/10.1016/j.carbpol.2015.02.042>

Lai, Y.-L., Lin, Y.-Y., Sadhasivam, S., Kuan, C.-Y., Chi, C.-y., Dong, G.-C., and Lin, F.-H. (2018). Efficacy of Bletilla striata polysaccharide on hydrogen peroxide-induced apoptosis of osteoarthritic chondrocytes. *Journal of Polymer Research* **25**. <https://doi.org/10.1007/s10965-018-1448-z>

Li, Y., Hao, Y., Yang, X., Zhao, J., Chang, R., Wang, B., and Zhan, X. (2025). Structure characterization of a Bletilla striata homogeneous polysaccharide and its effects on reducing oxidative stress and promoting wound healing in diabetic rats. *Int J Biol Macromol* **307**, 141904. <https://doi.org/10.1016/j.ijbiomac.2025.141904>

Li, Y., Ma, Z., Yang, X., Gao, Y., Ren, Y., Li, Q., Qu, Y., Chen, G., and Zeng, R. (2021). Investigation into the physical properties, antioxidant and antibacterial activity of Bletilla striata polysaccharide/chitosan membranes. *Int J Biol Macromol* **182**, 311-320. <https://doi.org/10.1016/j.ijbiomac.2021.04.037>

Liao, Z., Zeng, R., Hu, L., Maffucci, K. G., and Qu, Y. (2019). Polysaccharides from tubers of Bletilla striata: Physicochemical characterization, formulation of buccoadhesive wafers and preliminary study on treating oral ulcer. *Int J Biol Macromol* **122**, 1035-1045. <https://doi.org/10.1016/j.ijbiomac.2018.09.050>

Liu, C., Dai, K. Y., Ji, H. Y., Jia, X. Y., and Liu, A. J. (2022). Structural characterization of a low molecular weight Bletilla striata polysaccharide and antitumor activity on H22 tumor-bearing mice. *Int J Biol Macromol* **205**, 553-562. <https://doi.org/10.1016/j.ijbiomac.2022.02.073>

Luo, L., Fan, W., Qin, J., Guo, S., Xiao, H., and Tang, Z. (2023). Study on Process Optimization and Antioxidant Activity of Polysaccharide from Bletilla striata Extracted via Deep Eutectic Solvents. *Molecules* **28**. <https://doi.org/10.3390/molecules28145538>

Ma, D., Zhao, Z., Wen, Y., Zhou, J., Zhou, W., Mao, J., Lv, K., Cao, Y., and Jiang, L. (2025). The synergistic gelation of novel Bletilla striata polysaccharide with hyaluronic acid: Characterization, rheology. *Food Chem* **467**, 142359. <https://doi.org/10.1016/j.foodchem.2024.142359>

Ma, H., Axi, Y., Lu, Y., Dai, C., Huang, S., Kong, Z., Jimo, R., Li, H., Chen, G., Li, P., Zhang, L., Qu, Y., Qin, X., Zeng, R., and Gou, K. (2024). A dual network cross-linked hydrogel with multifunctional Bletilla striata polysaccharide/gelatin/tea polyphenol for wound healing promotion. *Int J Biol Macromol* **265**, 130780. <https://doi.org/10.1016/j.ijbiomac.2024.130780>

Qiu, J., Xu, X., Guo, J., Wang, Z., Wu, J., Ding, H., Xu, Y., Wu, Y., Ying, Q., Qiu, J., Wu, S., and Shi, S. (2024a). Comparison of extraction processes, characterization and intestinal protection activity of Bletilla striata polysaccharides. *Int J Biol Macromol* **263**, 130267. <https://doi.org/10.1016/j.ijbiomac.2024.130267>

Qiu, M., Zhong, G., Zhang, J., Hou, Y., Duan, Y., Guo, P., Jiang, F., Gou, K., Zhang, C., and Qu, Y. (2024b). Biocompatible and biodegradable Bletilla striata polysaccharides hydrogels crosslinked by BDDE for wound healing through the regulating of macrophage polarization. *Int J Biol Macromol* **254**, 128015. <https://doi.org/10.1016/j.ijbiomac.2023.128015>

Qu, Y., Li, C., Zhang, C., Zeng, R., and Fu, C. (2016). Optimization of infrared-assisted extraction of Bletilla striata polysaccharides based on response surface methodology and their antioxidant activities. *Carbohydr Polym* **148**, 345-53. <https://doi.org/10.1016/j.carbpol.2016.04.081>

Thacker, M., Tseng, C. L., Chang, C. Y., Jakfar, S., Chen, H. Y., and Lin, F. H. (2020). Mucoadhesive Bletilla striata Polysaccharide-Based Artificial Tears to Relieve Symptoms and Inflammation in Rabbit with Dry Eyes Syndrome. *Polymers (Basel)* **12**. <https://doi.org/10.3390/polym12071465>

Wang, B., Zhang, H., Chen, L., Mi, Z., Xu, Y., Zhao, G., Liu, S., Lei, H., Wang, Z., and Niu, J. (2020). Extraction, purification, and determination of the gastroprotective activity of glucomannan from Bletilla striata. *Carbohydr Polym* **246**, 116620. <https://doi.org/10.1016/j.carbpol.2020.116620>

Wang, Q., Chen, H., Yin, M., Cheng, X., Xia, H., Hu, H., Zheng, J., Zhang, Z., and Liu, H. (2023). In vitro digestion and human gut microbiota fermentation of Bletilla striata polysaccharides and oligosaccharides. *Frontiers in Cellular and Infection Microbiology* **13**. <https://doi.org/10.3389/fcimb.2023.1105335>

Wang, Q., Hu, H., Xiong, L., Jin, X., Zhu, T., Sun, X., Zhang, Z., and Liu, H. (2024a). Structural characterization and prebiotic activity of Bletilla striata polysaccharide prepared by one-step fermentation with Bacillus Licheniformis BJ2022. *Int J Biol Macromol* **258**, 128822. <https://doi.org/10.1016/j.ijbiomac.2023.128822>

Wang, S., Yu, J., Liu, Y., Yu, J., Ma, Y., Zhou, L., Liu, X., Liu, L., Li, W., and Niu, X. (2024b). Bletilla striata polysaccharide attenuated the progression of pulmonary fibrosis by inhibiting TGF-β1/Smad signaling pathway. *J Ethnopharmacol* **323**, 117680. <https://doi.org/10.1016/j.jep.2023.117680>

Wang, Y., Han, S., Li, R., Cui, B., Ma, X., Qi, X., Hou, Q., Lin, M., Bai, J., and Li, S. (2019). Structural characterization and immunological activity of polysaccharides from the tuber of Bletilla striata. *Int J Biol Macromol* **122**, 628-635. <https://doi.org/10.1016/j.ijbiomac.2018.10.201>

Wang, Y., Liu, D., Chen, S., Wang, Y., Jiang, H., and Yin, H. (2014). A new glucomannan from Bletilla striata: Structural and anti-fibrosis effects. *Fitoterapia* **92**, 72-78. <https://doi.org/10.1016/j.fitote.2013.10.008>

Xu, J., Chen, Z., Liu, P., Wei, Y., Zhang, M., Huang, X., Peng, L., and Wei, X. (2021). Structural characterization of a pure polysaccharide from Bletilla striata tubers and its protective effect against H(2)O(2)-induced injury fibroblast cells. *Int J Biol Macromol* **193**, 2281-2289. <https://doi.org/10.1016/j.ijbiomac.2021.11.060>

Zhai, W., Wei, E., Li, R., Ji, T., Jiang, Y., Wang, X., Liu, Y., Ding, Z., and Zhou, H. (2021). Characterization and Evaluation of the Pro-Coagulant and Immunomodulatory Activities of Polysaccharides from Bletilla striata. *ACS Omega* **6**, 656-665. <https://doi.org/10.1021/acsomega.0c05171>

Zhang, C., He, Y., Chen, Z., Shi, J., Qu, Y., and Zhang, J. (2019). Effect of Polysaccharides from Bletilla striata on the Healing of Dermal Wounds in Mice. *Evid Based Complement Alternat Med* **2019**, 9212314. <https://doi.org/10.1155/2019/9212314>

Zhang, J., Zhong, C., Chen, L., Luo, Y., Tang, L., Yang, J., Jia, J., Xie, X., Liu, P., Yu, J., and Cui, Y. (2025). Bletilla striata polysaccharide-mediated trained immunity drives the hematopoietic progenitors' expansion and myelopoiesis. *Int Immunopharmacol* **146**, 113909. <https://doi.org/10.1016/j.intimp.2024.113909>

Zhang, M., Sun, L., Zhao, W., Peng, X., Liu, F., Wang, Y., Bi, Y., Zhang, H., and Zhou, Y. (2014). Cholesteryl-modification of a glucomannan from Bletilla striata and its hydrogel properties. *Molecules* **19**, 9089-100. <https://doi.org/10.3390/molecules19079089>

Zhao, Y., Wang, J., Zhang, Y., Liu, C., Chen, Y., Li, P., Xu, T., Gao, L., and Zhang, W. (2025). Bletilla striata polysaccharide-based dissolving microneedle patch integrated with nanoparticles for promoting hair regrowth. *Int J Biol Macromol* **303**, 140336. <https://doi.org/10.1016/j.ijbiomac.2025.140336>

Zhao, Y., Wang, Q., Yan, S., Zhou, J., Huang, L., Zhu, H., Ye, F., Zhang, Y., Chen, L., Chen, L., and Zheng, T. (2021). Bletilla striata Polysaccharide Promotes Diabetic Wound Healing Through Inhibition of the NLRP3 Inflammasome. *Front Pharmacol* **12**, 659215. <https://doi.org/10.3389/fphar.2021.659215>

Zhong, G., Qiu, M., Zhang, J., Jiang, F., Yue, X., Huang, C., Zhao, S., Zeng, R., Zhang, C., and Qu, Y. (2023). Fabrication and characterization of PVA@PLA electrospinning nanofibers embedded with Bletilla striata polysaccharide and Rosmarinic acid to promote wound healing. *Int J Biol Macromol* **234**, 123693. <https://doi.org/10.1016/j.ijbiomac.2023.123693>

Zhu, J., Guo, X., Guo, T., Yang, Y., Cui, X., Pan, J., Qu, Y., and Wang, C. (2018). Novel pH-responsive and self-assembled nanoparticles based on Bletilla striata polysaccharide: preparation and characterization. *RSC Adv* **8**, 40308-40320. <https://doi.org/10.1039/c8ra07202g>
